# Dual Roles of Two Isoforms of Autophagy-related Gene *ATG10* in HCV-Subgenomic replicon Mediated Autophagy Flux and Innate Immunity

**DOI:** 10.1038/s41598-017-11105-3

**Published:** 2017-09-12

**Authors:** Qiong Zhao, Zhan-Ying Hu, Jing-Pu Zhang, Jian-Dong Jiang, Yuan-yuan Ma, Jian-rui Li, Zong-gen Peng, Jin-hua Chen

**Affiliations:** 1 Laboratory of pharmacology, Institute of Medicinal Biotechnology, Chinese Academy of Medical Sciences and Peking Union Medical College, Beijing, 100050 China; 2 State Key Laboratory of Bioactive Substances and Functions of Natural Medicines, Institute of Materia Medica, Chinese Academy of Medical Sciences and Peking Union Medical College, Beijing, 100050 China

## Abstract

Autophagy and immune response are two defense systems that human-body uses against viral infection. Previous studies documented that some viral mechanisms circumvented host immunity mechanisms and hijacked autophagy for its replication and survival. Here, we focus on interactions between autophagy mechanism and innate-immune-response in HCV-subgenomic replicon cells to find a mechanism linking the two pathways. We report distinct effects of two autophagy-related protein ATG10s on HCV-subgenomic replication. ATG10, a canonical long isoform in autophagy process, can facilitate HCV-subgenomic replicon amplification by promoting autophagosome formation and by combining with and detaining autophagosomes in cellular periphery, causing impaired autophagy flux. ATG10S, a non-canonical short isoform of ATG10 proteins, can activate expression of IL28A/B and immunity genes related to viral ds-RNA including *ddx-58*, *tlr-3*, *tlr-*7, *irf-3* and *irf-7*, and promote autophagolysosome formation by directly combining and driving autophagosomes to perinuclear region where lysosomes gather, leading to lysosomal degradation of HCV-subgenomic replicon in HepG2 cells. ATG10S also can suppress infectious HCV virion replication in Huh7.5 cells. Another finding is that IL28A protein directly conjugates ATG10S and helps autophagosome docking to lysosomes. ATG10S might be a new host factor against HCV replication, and as a target for screening chemicals with new anti-virus mechanisms.

## Introduction

Autophagy and immune response are two defense mechanisms that human body uses against viral infection. Autophagy is well known as an important cellular mechanism by which cells maintain homeostasis by degrading and recycling long-living proteins and cellular organelles, clearing pathogens such as bacteria and viruses, and regulating the immune system. The human immune system defends the body by sensing pathogens, such as viruses and bacteria, activating a series of immune responses and cytokines, and clearing pathogens in host cells. Nevertheless, in certain people, the two defense systems are too weak to eradicate persistent hepatitis C virus infection from human cells. In clinical settings, more than 70% of HCV patients develop sustained intrahepatic infection, fibrosis, cirrhosis, or hepatocellular carcinoma^1^, indicating the immune function and the autophagy mechanism nearly failed in defending HCV chronic infection. Previous studies have documented that some viral mechanisms can exploit host resources, circumvent host immunity mechanisms, and hijack the autophagy network for its own replication and survival^2–5^. Autophagy helps HCV survive in at least two ways: providing an autophagosome membrane network upon which HCV may replicate^2, 6–9^, and disturbing the host immune system^10–13^ to prevent the virus from being detected and subjected to an immune response from the host. Both may be due to non-autophagic functions of some autophagy-related proteins such as beclin1, LC3B, Atg4B, Atg5, Atg7, and Atg12^7, 14^. Type III interferons that have been found in recent decades have received large amounts of attention because they have more specific and stronger anti-HCV roles and lower rates of extrahepatic adverse effects than IFN-α in patients with chronic HCV infection and *in vitro* studies^15, 16^, but their real mechanism still remains to be revealed. Are there still some silent factors in host hepatocytes that can be evoked to resist HCV replication? Is there some molecular mechanism to link the two defense systems? The purpose of this study was to explore these questions by focusing on interaction between HCV subgenome replication activity, the innate immune response and autophagy flux. For the first time, we report here distinct effects of two versions of autophagy-related protein ATG10 on HCV subgenomic replicon, which are involved in autophagy flux and innate immunity activity, particularly IL28A (IFN-III2, IFN-λ2).

## Results and Discussion

### Establishment of HCV-subgenomic replicon and NS5B action on autophagy induction

**Figure 1:**
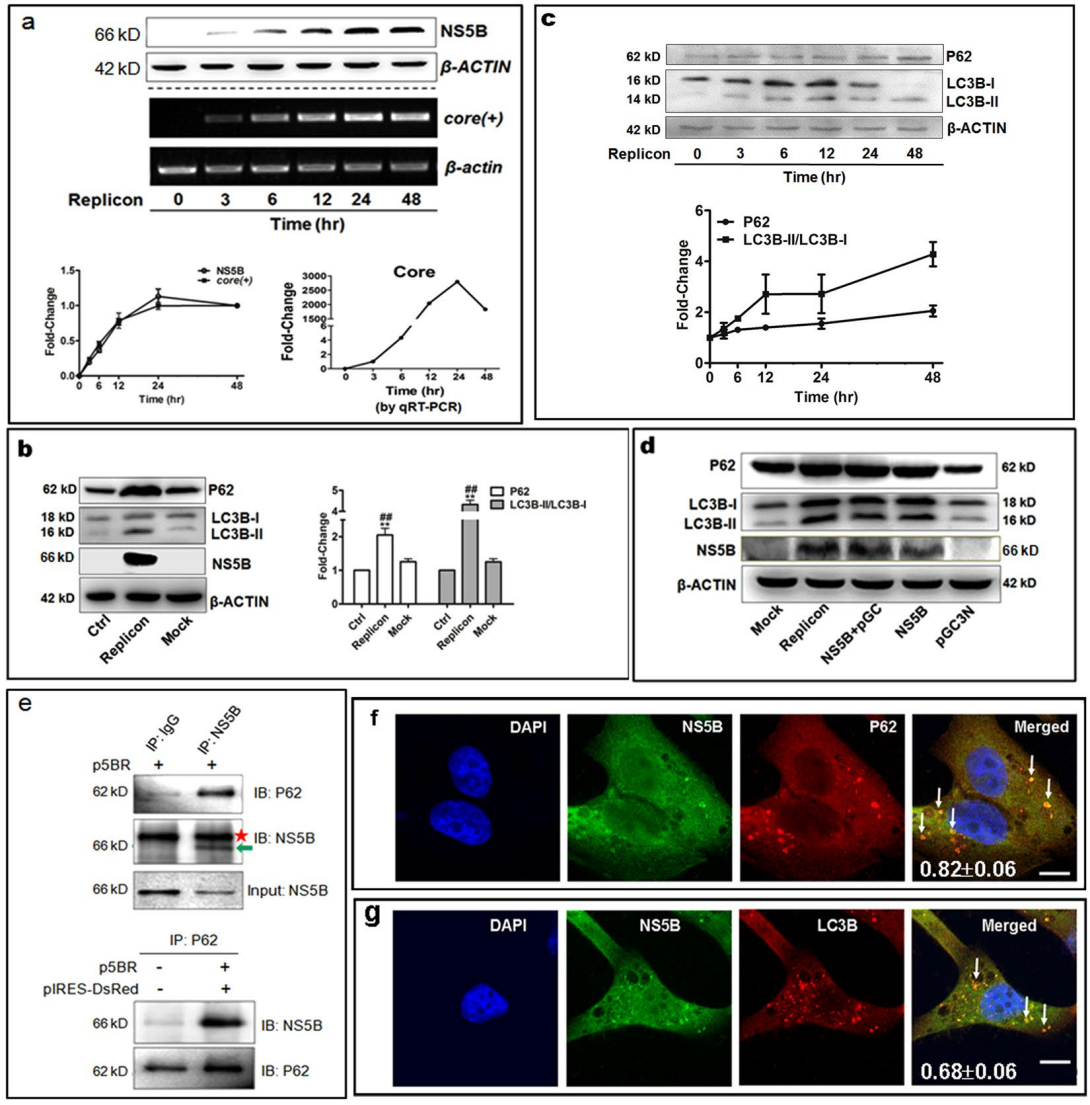
Establishment of HCV-subgenomic replicon and activation of autophagy by NS5B induction. (**a**) Time courses of *core* (+) levels and NS5B expression in HCV-subgenomic replicon cells detected by HCV-*core* dependent RT-PCR, quantitation real-time PCR and Western blot, respectively. (**b**) LC3B-II/I ratio and p62 protein levels at 48-h post-transfection. **P < 0.01 *vs* Control; ^##^P < 0.01 *vs* Mock. (**c**) LC3B-II/I ratio and p62 protein were increased in a time-dependent manner in HepG2 cells of the HCV-subgenomic replicon. (**d**) Elevation of LC3B-II/I ratio and P62 level were relied on NS5B expression. **(e)** Combination of NS5B protein with P62 was identified by Co-IP. The position of NS5B band is indicated by a green arrow, and non-specific bands indicated by a red star. (**f**,**g**) Co-localization of NS5B with p62 (**f**) and with LC3B (**g**) show HCV NS5B combined with autophagosomes (signed by white arrows) in the HCV subgenomic replicon cells. The values of Pearson coefficient and the standard deviation are showed at the bottom of the merged figures. Full-length or original blots/gels are presented in Supplementary Figure S6.

First, a cell model of HCV RNA subgenomic replicon was established using HepG2 cell line and the correlation between the HCV subgenomic replicon level and autophagy level was examined. The HCV subgenomic replicon consisted of two gene constructs, one expressing HCV RNA-dependent RNA polymerase (NS5B) and a red fluorescent protein gene separately with IRES fragment interval under a CMV promoter (here designated as p5BR); and the other can transcribe an antisense RNA molecule containing green fluorescent protein gfp and HCV 5′UTR-core, as well as sense HCV-3′UTR (here designated as pGC3N). The latter transcript served as a HCV RNA subgenomic template for simulating HCV RNA replication by HCV RNA-dependent RNA polymerase^17^. HepG2 cells were transfected with both p5BR and pGC3N and collected at an indicated time point. Tests of HCV-core dependent RT-PCR and qRT-PCR (here, core + level representing the HCV model replicative product) and Western blot analyses showed that levels of HCV-core RNA and the HCV RNA replicase NS5B increased in time-dependent manner, peaking between 24 h and 48 h post transfection (Fig. 1a), indicating that replication capability of the HCV subgenomic replicon is positively correlated to NS5B level and that the HCV subgenomic replicon model had been established successfully. The subgenomic replicon cells at 24 h and 48 h were chosen for further study.

Then the autophagy level and HCV subgenomic replicon level were assessed to determine if they were correlated, because autophagy is involved in viral replication^18, 19^. Western blotting results showed that autophagy marker LC3B-II/I protein ratio and selective adaptor p62 protein level were significantly higher in the replicon than in the control (GH cells) and the mock at 48 h post-transfection (Fig. 1b). High levels of p62 with high ratios of LC3B-II/I indicated an incomplete or defective autophagy process^20–23^. Whether this phenomenon is related to the potency of the model replication remains to be seen. A time-course test confirmed that both LC3B-II/I ratio and p62 protein level also increased gradually as the HCV-core level and NS5B protein level increased (Fig. 1a and c). This evidence suggests that the HCV subgenomic replication probably promoted autophagosome formation but impaired the autophagy flux. Further, we detected if NS5B alone could activate autophagy process using couples of HCV gene constructs. HepG2 cells were separately transfected with plasmid p5BR only, mixture of pGC3N and NS5B (the replicon), mixture of pGC and NS5B, pGC only and the mock. Plasmid pGC is derived from pGC3N with deletion of HCV 3′UTR sequence, which is replication defective. The results showed that elevation of LC3B-II/I ratio and p62 protein levels were detected in the cells transfected with p5BR alone, the HCV replicon, and mixture of pGC and NS5B, in which NS5B expression construct was included (Fig. 1d). These results suggest that activation of autophagy only relies on NS5B protein in the HCV subgenomic replicon cells.

Then, whether can NS5B protein be recognized and combined by selective adaptor p62? A Co-IP test used to show that NS5B directly combined with p62 protein (Fig. 1e). Again, using laser confocal microscopy and cell double immunofluorescence staining, the results presented two kinds of co-localized particles, HCV-NS5B protein incorporating with p62 and HCV-NS5B protein incorporating with LC3B (Fig. 1f and g), confirming that NS5B was bond by p62 and combined with the autophagosomes. Further, interaction of NS5B with P62 was examined in Hu 7.5 cells with infective HCV virion. Similarly, co-localized particles of NS5B incorporated with P62 were observed (see Supplementary Figure S5d). Thus, it is here supposed that HCV NS5B may induce autophagy but block autophagy flux to protect the HCV subreplicon from autophagic degradation.

### Distinct roles of two isoforms of autophagy-related protein ATG10 act on HCV subgenomic replicon activity and autophagy flux

**Figure 2:**
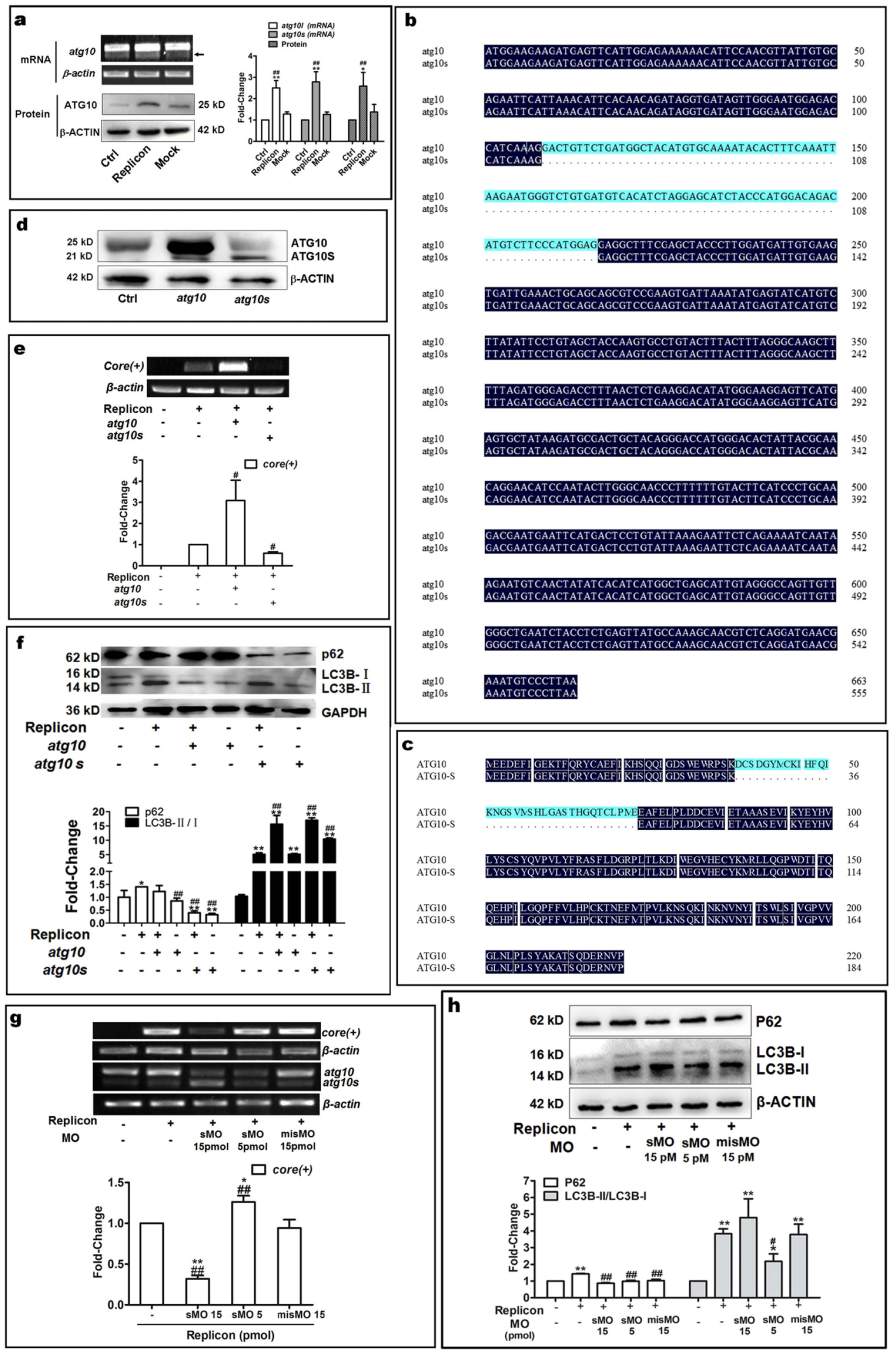
Expression and distinct roles of two isoforms of ATG10 proteins on HCV replication and autophagy flux in HepG2 cells. (**a**) ATG10 levels of mRNA and protein are elevated in the HCV subgenomic replicon cells. An arrow indictes *atg10s* band. The histograms show the relative levels of *ATG10* mRNA, *ATG10s* mRNA and ATG10 protein. *P < 0.05, **P < 0.01 *vs* Control; ^#^P < 0.05, ^##^P < 0.01 *vs* Mock. Comparison of coding region sequences **(b)** and putative amino acid sequences **(c)** between Atg10 and Atg10s are in alignment. **(d)** Overexpression of ATG10 and ATG10S was verified by Western blotting. (**e**) HCV-*core* was elevated by *atg10* and suppressed by *atg10s* overexpression in the HCVsubgenomic replicon cells (replicon). ^#^P < 0.05 *vs* HCV subgenomic replicon. (**f**) P62 and LC3B-II/I are elevated in the replicon and the replicon plus *atg10*; p62 is suppressed and LC3B-II/I enhanced by *atg10s* in replicon and non-replicon cells compared to the replicon alone. *P < 0.05, **P < 0.01 *vs* Control; ^#^P < 0.05, ^##^P < 0.01 *vs* HCV subgenomic replicon. (**g**) Endogenous *atg10s* is elevated and HCV *core* suppressed by sMO-*atg10* (15 pmole) notably in the HCV subgenomic replicon. The mock of misMO does not affect endogenous *atg10/atg10s* transcription and HCV-*core*. *P < 0.05, **P < 0.01 *vs* misMO; ^##^P < 0.01 *vs* the replicon. (**h**) P62 is decreased and LC3B-II/I not notably changed by sMO-*ATG10* in the replicon. *P < 0.05, **P < 0.01 *vs* control; ^#^P < 0.05, ^##^P < 0.01 *vs* the replicon. Full-length or original blots/gels are presented in Supplementary Figure S7.

Our results demonstrated that NS5B might activate autophagy, so we tested some autophagy-related proteins which play at the early stage of autophagy process, such as ATG3, ATG5, ATG6, ATG10 and so on; among them ATG10 expression changed evidently. The ATG10 transcriptional and translational levels were found to be significantly enhanced in the HCV subgenomic replicon cells (Fig. 2a), indicating that ATG10 may be involved in replication of the HCV virus. Further, we transfected p5BR into HepG2 cells and detected ATG10 protein by western blotting, which showed clearly that level of ATG10 protein was increased by alone NS5B expression as by the replicon (see Supplementary Figure S1). Fortunately, two variants of ATG10 gene were unexpectedly identified by RT-PCR; the longer band was predominant and the shorter one was nearly undetectable (here called ATG10 and ATG10S, respectively) in the HCV subgenomic replicon model, control, and mock groups (Fig. 2a). ATG10 protein is a canonical and has been documented as an E2-like enzyme to participate in catalysis of ATG12-ATG5 conjugate at stage of autophagy nucleation in the common autophagy process^24–26^. The short isoform ATG10S was only derived from computational prediction referring to GenBank records, and has not demonstrated by experiments and no reports were involved in ATG10S function before this study. To investigate function of ATG10 and ATG10S in HCV replication, we cloned the both variants of ATG10 full CDSs from HepG2 cell line. Sequencing and sequence comparison analysis were done in CDS and putative amino acid sequences (Fig. 2b,c). The results show that ATG10 is in concordance with human autophagy related 10 transcript variant 3 and ubiquitin-like-conjugating enzyme ATG10 (GenBank NM_001131028.1), and ATG10S is identical to human autophagy related 10 (ATG10) transcript variant X3 and ubiquitin-like-conjugating enzyme ATG10 isoform X3 (GenBank XM_005248612.1) (also reference to GenBank NCBI Reference Sequence: NC_000005.10). ATG10 CDS is consisted of 663 nucleotides which encode 220 amino acids and ATG10S CDS has 555 nucleotides which encode 184 amino acids. There is deletion of 36 amino acids in the N-terminal of ATG10S, which was encoded in exon-4 of atg10 transcript variant 3 sequence in human chromosome 5. Except the 36 amino acid difference, the rest sequences are identical between the two isoforms. Subsequently, ATG10 and ATG10S eukaryotic expression vectors were constructed in pIRES2-EGFP. Transfection and Western blotting experiments confirmed that both ATG10 vectors expressed the correct ATG10 proteins in HepG2 cells (Fig. 2d). Then ATG10 and ATG10S expression vectors were transfected into HCV subgenomic replicon cells, and the HCV subgenomic replicon level was examined using HCV-core (+) RNA-dependent RT-PCR. The results showed that overexpression of ATG10S significantly suppressed HCV duplication, but ATG10 strongly increased the HCV replicon level over that observed in the HCV replicon alone cells (Fig. 2e). The two isoforms of ATG10 proteins were found to have distinct roles on HCV subgenome replication.

Next, the autophagy level in the HCV subgenomic replicon cells was assessed to determine whether it could also be affected by overexpression of the two isoforms of ATG10. Western blot analysis showed the LC3B-II/I ratio to be significantly elevated in the HCV subgenomic replicon, the replicon plus ATG10, the replicon plus ATG10S, ATG10 alone, and ATG10S alone overexpression groups. The level of p62 protein was visibly decreased by ATG10S and not by ATG10 in HCV subgenomic replicon cells, relative to the replicon alone group; both ATG10 alone groups show the similar levels to the replicon plus ATG10 or plus ATG10S groups, respectively (Fig. 2f). Then, we used a gene knockdown method to verify these results. A morpholino oligo (ATG10-sMO, or sMO) can switch ATG10 to ATG10S product by removing exon-4 in ATG10 mRNA, so this oligo was transfected into HCV subgenomic replicon cells. As expected, ATG10 mRNA was down-regulated and ATG10S mRNA up-regulated relative to the control, the replicon, and misMO-transfected cells in the group with sMO, simultaneously, HCV-core level was significantly lower in the HCV replicon cells subjected to ATG10-sMO treatment than in the control and misMO groups (Fig. 2g), confirming that host endogenous ATG10S elevation can also efficiently suppress duplication of the HCV subgenomic replicon. P62 protein and LC3B-II/I ratio underwent the similar changes upon sMO (at 15 pmol) treatment as in the HCV subgenomic replicon given ATG10S overexpression: p62 was degraded and LC3B-II/I elevated (Fig. 2h). Meanwhile, we also observed opposite on the level of HCV-core at the low concentration (5 pmol) of sMO, in which *atg10* mRNA level reduced, but *atg10s* mRNA did not changed due to some unknown causes; correspondingly, core level increased moderately and LC3BII/I rate decreased compared with the replicon group (Fig. 2h). These evidences hint that ATG10 at low level may disturb maturation of autophagosomes by its inefficient E2-like function, leading to incomplete autophagy process and mild increase of HCV subreplicon too. It is here inferred that ATG10S protein probably promotes breakdown of HCV RNA subgenomic replisome via recovering the complete autophagy flux.

### Innate immune responses are regulated differently by ATG10 two versions

**Figure 3:**
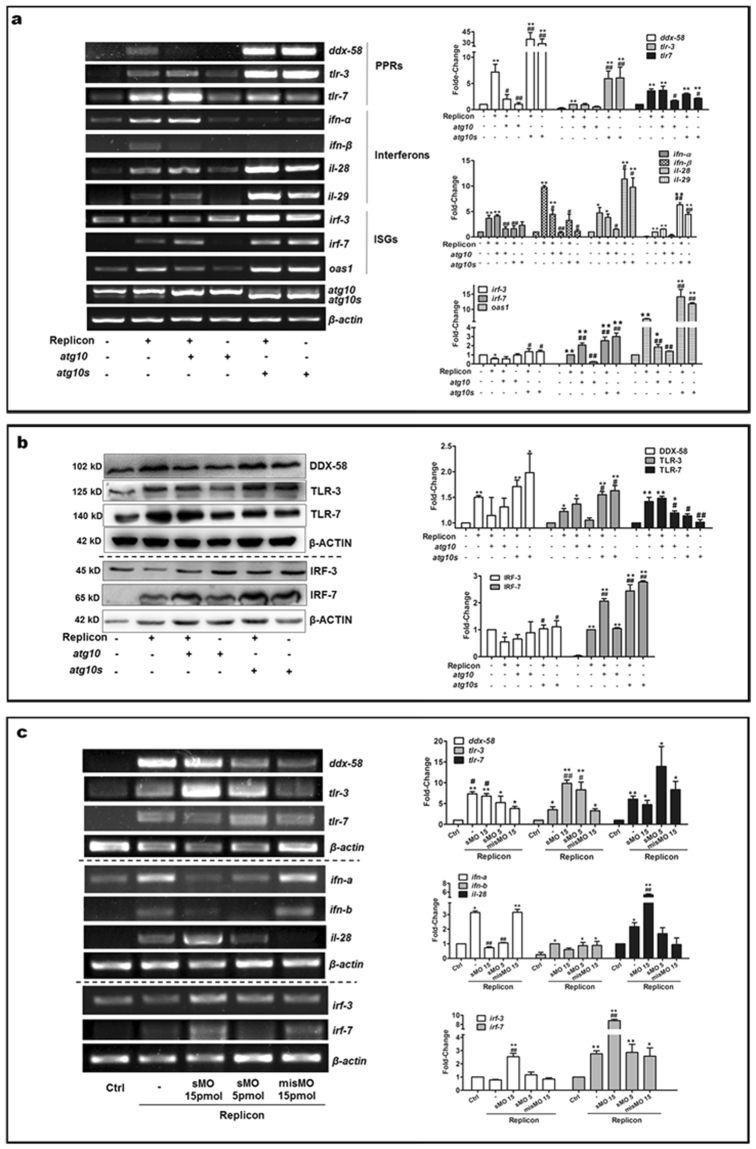
Differential regulation of two ATG10 isoforms on host innate immunity in the HCV-subgenomic replicon cells. HepG2 cells were transfected with the HCV subreplicon or with the subreplicon plus ATG10/ATG10S, or transfected with only ATG10/ATG10S, and collected after an additional 48 h of culture for detection of innate immunity related gene expression. (**a**) Transcription levels of PRRs (*ddx-58*, *tlr-3* and tlr-*7*), IRFs (*irf-3, irf-7*and *oas1*) and IFNs (*ifn-α*, *ifn-β*, *il-28* and *il-29*) are differential regulated by overexpression of *atg10s* and *atg10*. *P < 0.05, **P < 0.01 *vs* Ctrl; ^#^ *P* < 0.05, ^##^ *P* < 0.01 *vs* the replicon. (**b**) Proteins of DDX-58, TLR-3/7, and IRF-3/-7 are regulated by overexpression of *atg10s* and *atg10*. *P < 0.05, **P < 0.01 *vs* control; ^#^ *P* < 0.05, ^##^P < 0.01 *vs* the subreplicon. (**c**) The transcription levels of the innate immune factors, tlr-3/-7, IRF-3/-7, type I and type III IFNs are regulated by sMO-*atg10*. *P < 0.05, **P < 0.01 *vs* control; ^#^ *P* < 0.05, ^##^ *P* < 0.01 *vs* misMO. (n = 3). Full-length or original blots/gels are presented in Supplementary Figure S8.

Since both ATG10s can differentially affect HCV subgenomic replication, and HCV virus infection can strongly activate innate immunity^1, 27, 28^, we asked whether innate immunity also could be impacted differently by the two ATG10 isoforms. The expression of some innate immunity-related genes, such as interferons of type1 (*ifn-α* and *ifn-β*) and type3 (*il28a/b* and *il29*), pattern recognition receptors (PRRs), and interferon regulatory factors (*IRFs*), were assessed by RT-PCR and Western blot analysis, and compared their levels between the HCV subgenomic replicon and the replicon plus *ATG10S* or plus *ATG10* respectively. As shown in Fig. 3, the innate immune response was induced in the HCV subgenomic replicon cells (collected after 48 hours culture subsequent to transfection), including three *IFNs* (*ifn-α*, *ifn-β* and *ifn-λs*), three *PRRs* (*tlr-3*, *tlr-7*, and *ddx-58*) and *irf-7* were notably up-regulated; only *irf-3* was down-regulated (Fig. 3a and b). It is here proposed that the elevation of these genes may be caused by host instinctual innate immune response stimulated by the HCV subgenomic replicon activity. However, the HCV subgenomic replicon can selectively suppress IRF3 expression, an important inducer of interferon genes and a downstream signal of TLR3. Since TLR3 is an endosome membrane receptor for monitoring viral dsRNA, we postulate that the HCV subgenomic replicon can resist host innate immunity response via inhibiting IRF3 expression to block TLR3 signal.

In the group of HCV subgenomic replicon plus *ATG10*, most of these genes underwent changes similar to those observed in the subgenomic replicon cells, but *ifn-β*, *oas1* and *ddx-58*/DDX-58 showed in lower levels, and the *irf-7*/IRF-7 was significantly higher than in the replicon. In the group treated with *ATG10* overexpression alone, the levels of innate immune factors were similar to those observed in the control. However, in the HCV subgenomic replicon plus *ATG10S* group, alone *ATG10S* group, and the subgenomic replicon plus *ATG10*-sMO group (15 pmol/L), *il28a/b* and *il29*, *oas1*, *tlr-3*/TLR-3, *ddx-58*/DDX-58, *irf-3*/IRF-3, and *irf-7*/IRF-7 were more notably and selectively up-regulated than in the HCV subgenomic replicon, the replicon plus *ATG10*, and the replicon plus misMO group (at 15 pmol/L) (Fig. 3a–c). Five of these immunity proteins were verified by qRT-PCR, which showed the similar variation of *il28a*, *tlr3*, *tlr7*, *irf-3* and *irf-7* affected by overexpression of atg10 and atg10s (see Supplementary Figure S2) to the semi-RT-PCR results.

Previous works have demonstrated that autophagy is involved in the immune response^29–32^. This study shows that ATG10S has intensive roles in selective activation of HCV-dsRNA-correlated innate immunity factors, such as DDX-58, TLR3, IRF3, and IRF-7, of which, DDX-58 is a cytosol receptor for monitoring viral dsRNA and activating IRF3 and IRF7 functions. These four factors further trigger IFNs expression, particularly IFNλs expression^1, 30, 32, 33^. Besides, ATG10S can suppress TLR7 that functions in sensing viral ssRNA in the HCV subgenomic replicon cells. Thus, we infer that one of ATG10S functions may be to trigger specific innate immunity response targeting to viral ds-RNA via indirectly activating the sensors DDX-58 and TLR-3. ATG10 did not show the same roles as ATG10S on the immunity response, indicating that ATG10 may be implicated in different signaling pathways from ATG10S. As we know, immune regulation is complicated and involved in multiple signaling interactions including induction or inhibition of pathogenic organisms, interaction among immune factors and other host factors with pathogens, or negative feed-back regulation by the IFN/ISGs response, and so on. The two ATG10 proteins’ different mechanisms underlying the immune regulation remain to be explored.

### Both ATG10 and ATG10S can incorporate into autophagosomes and differentially impact autophagosome subcellular localization and autolysosome formation

**Figure 4:**
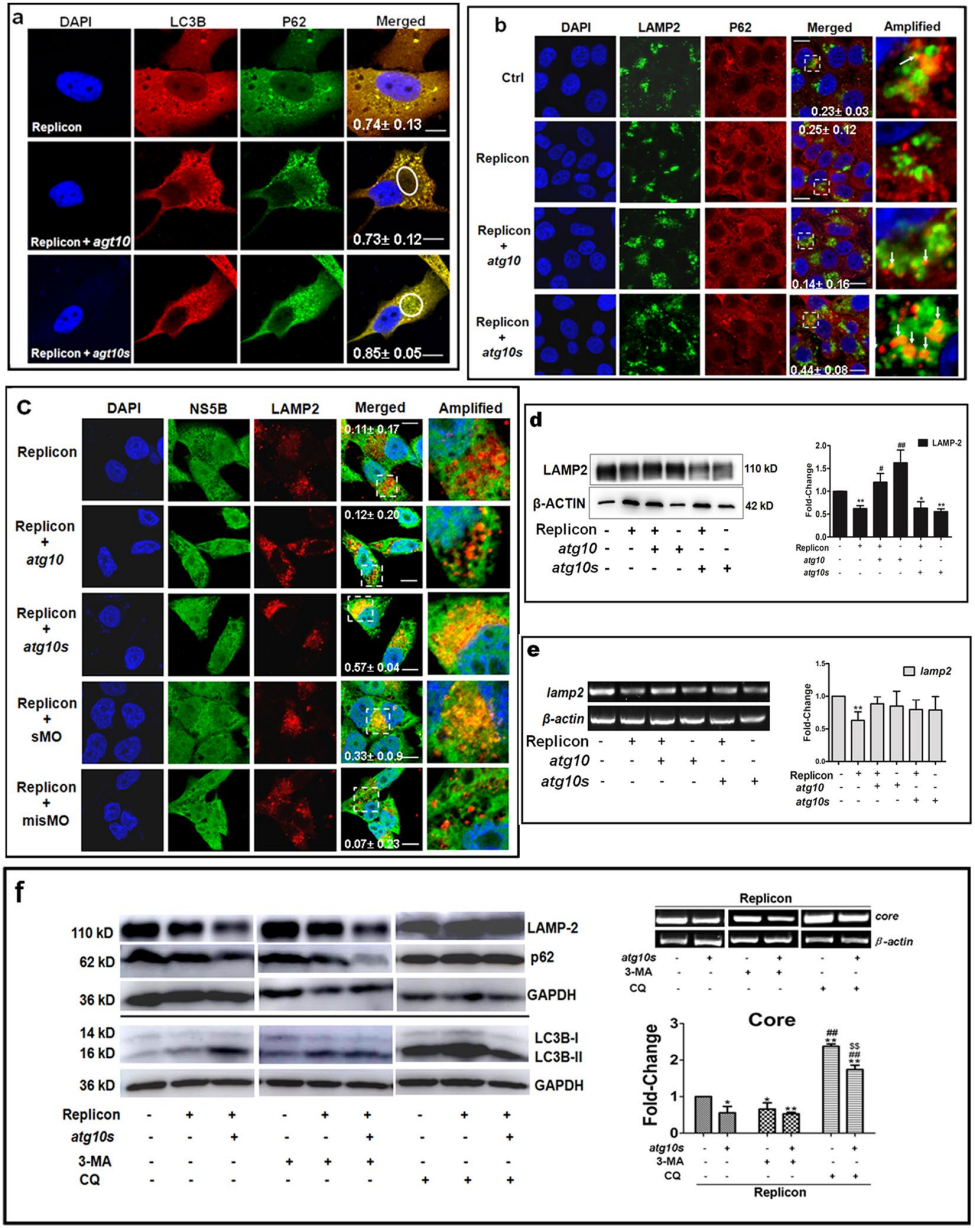
Subcellular localization of autophagosomes and formation of autolysosomes are differentially regulated by ATG10 and ATG10S in the HCV-subgenomic replicon cells. (**a**) ATG10 protein detains p62-LC3B conjugates (representing autophagosomes) at cell periplasm and ATG10S transports the autophagosomes centripetally to the perinuclear region. (**b**) Co-localization of p62 with LAMP2 (white arrows in the amplified pictures) is promoted by *atg10s* overexpression and not by *atg10* relative to the control and the replicon groups. Images in the dashed squares are amplified at the right pictures. (**c**) More co-localization of NS5B with LAMP2 is facilitated by *atg10s* and by sMO-*atg10* than by *atg10* and misMO in the HCV replicon cells. Images in the dashed squares are enlarged at the right pictures. The values of Pearson coefficient and the standard deviation are showed in the merged figures (in **a**–**c**). Scale bars = 10 μm. (**d**) Western blotting shows LAMP2 level elevated in both *atg10* groups but reduced in the HCV replicon and both *atg10s* groups compared to the control group. *P < 0.05, **P < 0.01 *vs* ctrl; ^#^ *P* < 0.05, ^##^ *P* < 0.01 *vs* the replicon. (**e**) RT-PCR indicates lower level of *lamp2* mRNA in the subreplicon group than in the control group and the other four groups. **P < 0.01 *vs* ctrl. (**f**) Influence of autophagy inhibitors on ATG10S function in autophagy flux and HCV subreplicon. 3-MA alone moderately decreased HCV-core level but did not affect autophagy flux apparently; when ATG10S overexpressed, LAMP2 and p62 declined and LC3B-II/I ratio elevated, and HCV replication was suppressed obviously compared to the replicon group. CQ treatment significantly elevated HCV-core level and caused accumulation of autolysosomes compared to the replicon group though overexpression of *atg10s* relatively decreased HCV-core level in the group of replicon plus CQ. *P < 0.05, **P < 0.01 *vs* the replicon alone; ^##^ *P* < 0.01 *vs* the replicon plus *atg10s*; ^$$^ *P* < 0.01 *vs* the replicon plus CQ. All data are mean ± sd (n = 3). Full-length or original blots/gels are presented in Supplementary Figure S9.

Based on our new findings that show that ATG10S can recover the autophagy flux blocked by the HCV subgenomic replicon and activate innate immunity-related factors, the differential mechanisms underlying the two isoforms of ATG10 involved in the HCV-xenophagy were investigated further. Using the laser confocal microscopy and cell double immunofluorescence staining, we found that, in the HCV subgenomic replicon cells, ATG10 overexpression caused p62-LC3B colocalized particles to become distributed at the cellular periphery (Fig. 4a). In this way, a perinuclear “restricted zone” was established by ATG10; but ATG10S overexpression drove p62-LC3B colocalized particles aggregated to the perinuclear “restricted zone”, as compared to the HCV replicon group in which there were a few irregular p62-LC3B particles scattered freely in the cytoplasm (Fig. 4a). Though number and distribution of the colocalized particles were variant, the p62-LC3B conjugation showed similarly high Pearson coefficient values (>0.7) in the three groups with or without ATG10/ATG10S in the replicon cells (see Supplementary Figure S3a). It is not clear which subcellular organelle settle in the “restricted zone”. The subcellular localization of p62-cargo and LAMP2, one of lysosomal membrane proteins, was examined among the control, the replicon, the replicon plus *ATG10*, and the replicon plus *ATG10S* groups. Most of LAMP2 particles gathered at the perinuclear region in the “restricted zone” in all four groups, which show that it is the lysosomes that settle in the “restricted zone”. Importantly, colocalized particles of p62-cargo and LAMP2 emerged clearly in the replicon plus *atg10s* group and not in the other three groups (Fig. 4b and Supplementary Figure S3b), which means ATG10S overexpression can drive autophagosomes to dock to lysosomes in the HCV subreplicon cells.

Another colocalization experiments was performed using NS5B as the HCV subgenomic replicon tracer and LAMP2, and produced the same results: most of the NS5B-LAMP2 conjunct particles were detected in the perinuclear region in the replicon plus *atg10s* group; and independent dots of NS5B or LAMP2 in mosaic distribution and few colocalized granules appeared loosely in the replicon and the replicon plus *atg10* groups (Fig. 4c and Supplementary Figure S3c). These results were confirmed using a splicing morpholino oligo (sMO*atg10*) that can switch *atg10* mRNA to *atg10s* mRNA, and more NS5B-LAMP2 conjoined particles reappeared in the perinuclear region than in the misMO control. In the misMO cells, particles of NS5B or LAMP2 independently distributed as in the HCV subgenomic replicon cells (Fig. 4c and Supplementary Figure S3c). Both exogenous *atg10s* overexpression and endogenous *atg10s* mRNA elevation can facilitate formation of autophagolysosomes that contain HCV NS5B.

Additionally, the recovery of the autophagy flux was verified by testing LAMP2 levels. LAMP2 protein was significantly lower in the replicon, the replicon plus *atg10s*, and *atg10s* alone groups, but increased visibly in the replicon plus *ATG10* and *atg10* alone groups relative to in the replicon alone using western blot analysis (Fig. 4d). Then, whether the decrease in LAMP2 was caused by transcriptional inhibition or autolysosomal degradation was examined. RT-PCR testing indicated significantly lower *lamp2* mRNA in the HCV subgenomic replicon than in the control but only slightly less in the other groups (Fig. 4e). The results demonstrate that the decrease of LAMP2 protein in the HCV replicon was caused by inhibition of *lamp2* gene transcription, which decreases the risk of HCV lysosomal degradation due to reduction of the lysosome number. The LAMP2 decrease in both *ATG10S* groups is mainly resulted from autophagolysosome degradation; and LAMP2 levels were elevated moderately in both ATG10 groups.

Further, which phase in autophagy process affected by ATG10S was investigated using autophagy inhibitors 3-methyladenine (3-MA) and chloroquine (CQ). We did not find differential change of LAMP2 protein and p62 protein between the two groups of the replicon with or without 3-MA; but LC3B-II/I ratio increased slightly and HCV replication was moderately inhibited in the replicon plus 3-MA. Notably, when ATG10S overexpressed in the replicon plus 3-MA group, LAMP2 and p62 declined and LC3B-II/I ratio elevated, and accordingly HCV replication was suppressed obviously compared to the replicon group, but the inhibition role on the HCV replicon was similar to that in the replicon plus ATG10S group (Fig. 4f). So we infer that effects of 3-MA and ATG10S in the xenophagy are acting on different targets. In contrast, when the cells treated by CQ, levels of LAMP2, P62 and LC3B-II/I were totally elevated in the three groups including the CQ-contained control group compared to the replicon and control groups (Fig. 4f). The core level is significantly elevated in the replicon plus CQ with or without ATG10S compared to the replicon alone (Fig. 4f). As we know, 3-MA inhibition occurs at the phase of isolation membrane in autophagy, which blocks extension and maturation of autophagic vacuoles, leading to decrease of endomembrane amount and suppression of the HCV subreplicon. CQ functions in blocking formation of autolysosomes at the late stage of autophagy, so that duplication of HCV subreplicon is not suppressed but enhanced greatly due to increase of cellular endomembrane amount and inhibition of lysosomal degradation. Since ATG10S inhibition on the HCV replicon was predominately prevented by CQ, we suppose that phase of ATG10S action is probable at autolysosome formation.

### Synergism of IL28A and ATG10S degrades HCV subgenomic replicon via linking autophagosomes to lysosomes and restoring autophagy flux

**Figure 5:**
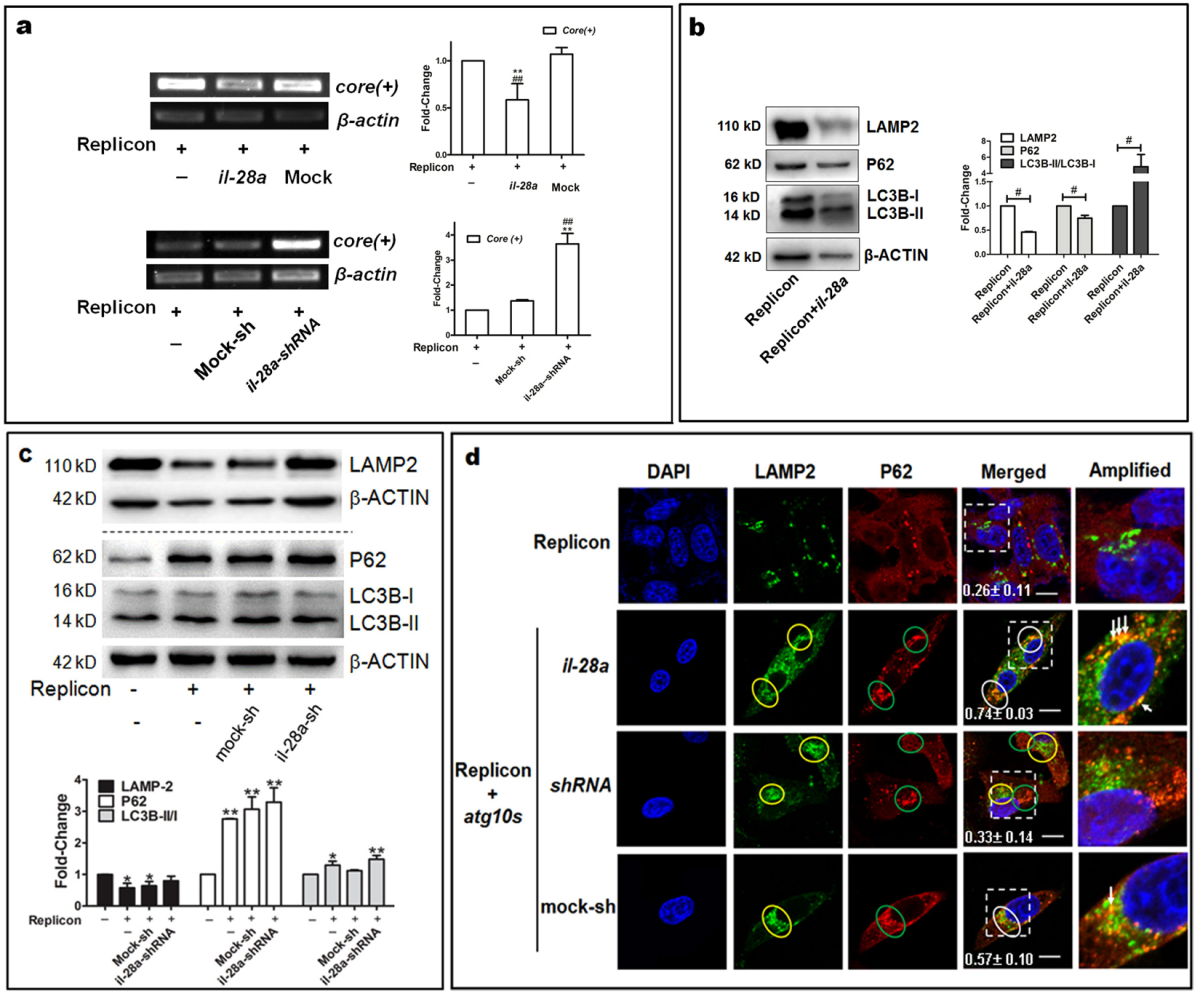
Synergistic action of IL28A with ATG10S inhibits HCV subgenome replication via restoration of autophagy flux. (**a**) HCV subgenomic replicon activity was suppressed by IL28A overexpression and enhanced by IL28A knockdown. **P < 0.01 *vs* the replicon; ^##^ *P* < 0.01 *vs* mock. (**b**) IL28A overexpression reduced LAMP2 and p62 protein levels but increased LC3B-II/I ratio relative to the replicon group. ^#^ *P* < 0.05 *vs* the replicon. (**c**) IL28A knockdown moderately elevated levels of LAMP2, P62 and LC3B-II/I ratio compared to the replicon group. *P < 0.05, **P < 0.01 *vs* control. (**d**) IL28A can mediate combination of autophagosomes with lysosomes (signed by white arrows in the amplified pictures) in the HCV replicon plus *atg10s*. IL28A downregulation blocks autolysosome formation by shRNA interference (green particles and red particles separated). Yellow circles show some of the accumulated lysosomes (LAMP2), green circles show some of the accumulated autophagosomes (p62), and white circles show some autolysosomes; images in the dashed squares are enlarged at the right pictures and white arrows indicate autolysosomes. The values of Pearson coefficient and the standard deviation are put in the merged figures. Scale bars = 10 μm. Full-length or original blots/gels are presented in Supplementary Figure S10.

The above results suggest that ATG10S probably has at least two pathways or mechanisms by which it may suppress HCV RNA amplification: restoring autophagy flux by promoting fusion of autophagosomes to lysosomes, and activation of the innate immune system, particularly dominant activation of *il28a/b* expression. Further, we investigated how these two mechanisms could act in coordination to stop HCV subgenome amplification and what roles IL28A played in the process. First, it was determined whether IL28A could inhibit HCV replication. RT-PCR tests showed that *il28a* overexpression caused *core* (+) levels to decrease significantly compared to the mock and the replicon. In contrast, knockdown of *il28a* expression by shRNA reversed this result—the HCV-*core* level was much higher than in the replicon and the replicon plus shRNA mock groups (Fig. 5a). Clearly, IL28A play a role in the inhibition of the HCV subgenome replication.

Next, the manner in which IL28A performed the inhibition and any correlation to ATG10S in subcellular behavior was investigated. Western blot tests showed that *il28a* overexpression reduced levels of LAMP2, p62, and the total amount of LC3B but elevated the ratio of LC3B-II/I (Fig. 5b), indicating that autophagy flux is complete and the autolysosomes have been degraded in the HCV subgenomic replicon cells. Transfection of *IL28A* shRNA was associated with moderately higher levels of LAMP2 protein, P62 protein and LC3B-II/I ratio than in the HCV replicon (Fig. 5c), indicating that down-regulation of IL28A may enhance autophagosome accumulation and block lysosomal degradation. Recently, a study indicates that activity of IFN-λ1 anti-HCV was related to its action on down-regulating translation of ATG5 and GABARAP genes and inhibition of autophagosome formation^34^. Thus, we presume that two pathways or mechanisms of IL28A (IFN-λ2) may associate with its anti-HCV replication function. One is enhancement of autolysosomes formation by IL28A driving autophagosomes to lysosomes, which was proved in this study; and the other one may be partly involved in translational inhibition of autophagy-related genes including ATG5, LC3B and so on induced by IFNλs, which suppresses autophagosome formation at the nucleation and extension stages and decreases the endomembrane web (autophagosomes) where HCV replication situates on. P62 levels in the three groups replicon-contained were significantly higher than the control group, meaning that elevation of P62 level is correlated to the HCV subreplicon.

It was then determined if IL28A protein participated in the fusion of lysosomes to autophagosome. Cell immunofluorescence double staining showed that IL28A overexpression in the HCV replicon plus *atg10s* increased conjunct particles of LAMP2 and p62 which accumulated in the perinuclear region. Inversely, *il28a* knockdown by shRNA transfection caused LAMP2 and p62 to separate from each other but they still remained close to the nuclei in the HCV subgenomic replicon cells. No fused particles were detected in the HCV subgenomic replicon group, and there were fewer p62-cargo and lysosome granules dispersed independently (Fig. 5d and Supplementary Figure S3d). Based on these results we suppose that, with the help of IL28A, ATG10S can drive autophagosomes to combine with lysosomes; without *il28a* expression, autophagolysosomes were hard to be formed, even though ATG10S was found to drive the autophagosomesto the perinuclear space in the HCV replicon cells. In this way, IL28A maybe mediate ATG10S defense roles against HCV subgenome replication by drawing lysosomes and autophagosomes together and promoting lysosomal degradation. These results demonstrate that docking and fusion are two independent steps in autophagosome–lysosome fusion and that ATG10S may have a key function related to autophagolysosome formation.

### ATG10S directly combines to IL28A and fuses to autolysomes but ATG10 not

**Figure 6:**
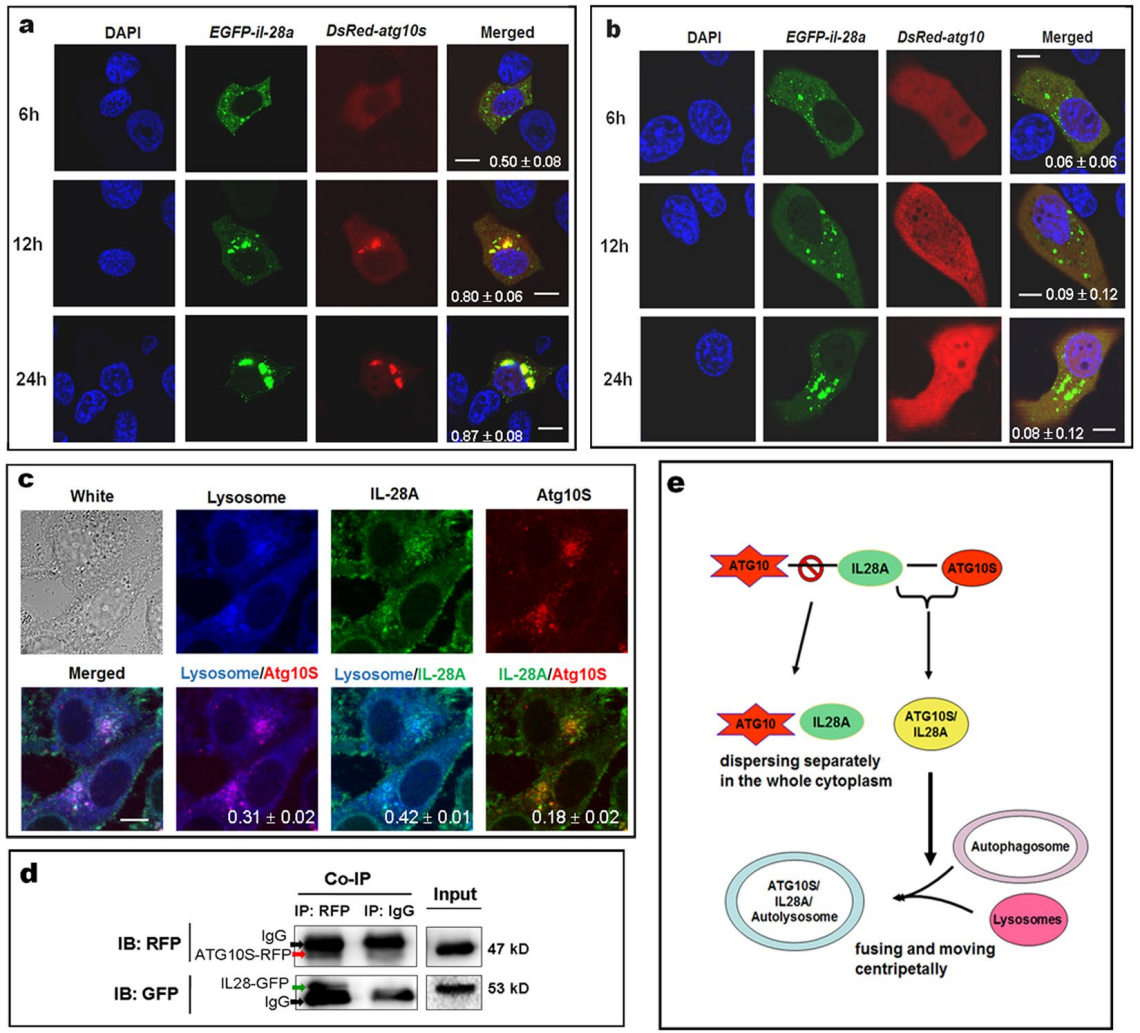
ATG10S combines to IL28A and fuses to autolysomes but ATG10 not. (**a**) Co-transfection of *egfp*-fused *il28a* expression plasmid and *DsRED*-fused *atg10s* plasmid shows that ATG10S had co-localized with IL28A, and the conjunct particles (yellow particles) grew and accumulated at perinuclear region in HepG2 cells in a time-dependent form. (**b**) Co-transfection of *egfp*-fused *il28a* plasmid and *DsRED*-fused *atg10* plasmid did not show IL28A-ATG10 conjugate in the whole experimental process. Scale bars = 10 μm (in **a** and **b**) **(c)** Using lysosome tracker dye, triple colocalization of lysosomes with IL28A and ATG10S was observed in HepG2 cells; two-by-two-merged figures show positive combined particles of lysosome-IL28A, lysosome-ATG10S, and IL28A-ATG10S, respectively. Scale bars = 8 μm (**c**). The values of Pearson coefficient and the standard deviation are put at the bottom of the merged figures (in **a**–**c**). (**d**) Co-IP test shows the interaction between IL28A and ATG10S using the *egfp*-fused *il28a* plasmid and *DsRED*-fused *atg10s* plasmid co-transfection in HepG2 cells. Immunoprecipitation with anti-RFP antibody showed combination of ATG10S with IL28A. A red arrow points to the fusion protein of ATG10S-RFP (about 47 kD), and a green arrow points to IL28 fused to GFP (about 53 kD). Black arrows indicate IgG protein (about 50 kD), which is an accompaniment of Immunoprecipitation. Full-length or original blots/gels are presented in Supplementary Figure S11d. (**e**) A model for IL28A interaction with two ATG10 proteins.

To investigate how IL28A mediated ATG10S function, using the same method and co-transfection of reporter-fused gene expression plasmids, pEGFP-IL28A and pDsRED-ATG10S or pDsRED-ATG10, the results showed that ATG10S indeed directly colocalized with IL28A in a high Pearson coefficient (see Supplementary Figure S3e), and the conjunct particles grew and accumulated in the perinuclear region in HepG2 cells in a time-dependent manner from 12 h after transfection (Fig. 6a). ATG10 protein did not colocalize with IL28A at any point during the experimental process (Fig. 6b and Supplementary Figure S3f). Further, co-localization of IL28 protein with ATG10S protein was confirmed using their endogenous IL-28 antibody and ATG10 antibody, which showed obviously the combination of IL28 with ATG10S (see Supplementary Figure S4). Using lysosome-tracker dye, experimental results indicated presentation of triple colocalization particles of lysosomes combined with IL28A and ATG10S; two-by-two-merged figures showed the conjunct particles of lysosome-IL28A, lysosome-ATG10S, and IL28A-ATG10S (Fig. 6c) with middle levels of the Pearson coefficients(see Supplementary Figure S3g), which might be partly impacted by the dye or some unknown factors.

Co-immunoprecipitation was used to verify the interaction between IL-28A and ATG10S. The positive result was shown by using anti-RFP antibody to co-precipitate IL28A protein in HepG2 cells which were co-transfected *egfp*-fused *il28a* expression plasmid and *DsRED*-fused *atg10s* plasmid (Fig. 6d). The results offer positive evidence that IL28A combines with ATG10S proteins, which may play a crucial linker role between autophagosomes and lysosomes. The ATG10S can promote transport of autophagosomes to lysosomes, where IL28A may help autophagosome docking to lysosomes. These findings are summarized in Fig. 6e which indicates possible synergetic mechanisms of ATG10 and IL28A proteins in HCV-xenophagy flux.

### Replication of infectious HCV virion can be inhibited by both ATG10 isoforms

Lastly, we examined both ATG10s’ inhibition effect on HCV replication in a full-length virus infection model. In this experiment, Huh7.5 cells were transfected with ATG10 or ATG10S full-length mRNA respectively and invaded with HCV virion (J6/JFH/JC). HCV RNA was reduced differentially by the two isoforms of ATG10 overexpression, but the ATG10S effect was more powerful than ATG10 and in dose-dependent manner (see Supplementary Figure S5a,a’) by qRT-PCR test. Meanwhile, HCV CORE and NS3 proteins were decreased significantly in dose-dependent manner by ATG10S overexpression and slightly by ATG10 using western blotting test (see Supplementary Figure S5b,b’). These results indicate that ATG10S might be efficient to suppress replication of HCV virion in the conventional virion infection cells, too. ATG10 action on the HCV infectious model presents different effect from on HCV subgenomic replicon, which, we presume, may be resulted from the different cell models.

We can infer that ATG10S may be a potential host-antiviral factor probably by activating host innate immunity systems and by rescuing impaired autophagy flux. Activation of ATG10S and IL28A may be suitable for the development of new therapeutic approaches to treating HCV and other RNA virus diseases. They may also serve as novel drug-targets with new anti-virus mechanisms for screening chemical candidates.

## Materials and Methods

### Reagents and antibodies

Lipofectamine 2000 Reagent was purchased from Invitrogen. shRNA (No. 32461-1) for IL28A downregulation and mock-shRNA were purchased from Shanghai Genechem Co. Protein extracting reagent RIPA lysis buffer (C1053) was purchased from Applygen Technologies, Inc. For Western blotting, anti-NS5B antibody (ab35586) was purchased from Abcam; anti-ATG10 (M151-3), anti-p62 (PM045), anti-LC3B (PM036) were purchased from MBL; another anti-ATG10 (A9356) antibody for both isoforms of ATG10 proteins was purchased from Sigma-Aldrich. Anti-LAMP2 (sc-18822) was purchased from Santa Cruz Biotechnology; Anti-TLR3 (6961), anti-DDX58 (4200), anti-TLR7 (2633), anti-IRF3 (11904), anti-IRF7 (4920) were purchased from Cell Signaling Technology (CST). Anti-β-ACTIN (TA09) and HRP-conjugated goat anti-mouse and goat anti-rabbit IgGs were purchased from ZSGB-BIO Co. (China). For immunoprecititation, anti-RFP mAb (M165-3) and anti-p62 (PM045) were purchased from MBL, anti-NS5B (ab35586) from Abcam, Rabbit IgG (A7016) and Mouse IgG (A7028) purchased from Beyotime Biotechnology.

For cellular immunofluorescence and co-localization experiments, anti-NS5B antibody (ab35586) and Human IL-28 antibody (ab38570) were purchased from Abcam. Anti-LC3B (M152-3), anti-p62 (M162-3) and anti-RFP mAb (M165-3) were purchased from MBL; anti-GFP (AM1009) was purchased from Abgent; anti-LAMP2 (sc-18822) and anti-hATG10 antibody (sc-70125) were purchased from Santa Cruz. The secondary antibodies TRITC-labeled goat anti-rabbit IgG, FITC-labeled goat anti-mouse IgG and mounting medium with DAPI (ZLI-9557) were purchased from ZSGB-BIO (China). LysoTracker Yellow HCK-123 was purchased from Invitrogen.

### HCV subgenomic replicon model and mock cells

In the transfection experiments, all the plasmids and shRNA were used at a concentration of 2.5 μg/well in 6-well plates, in accordance with the kit instructions provided with the Lipofectamine 2000 Reagent.

HepG2 cells were cultured and maintained in MEM (Gibco) containing 10% FBS (Hyclone). HepG2 cells were transfected with plasmid prGC3N using Lipofectamine 2000 Reagent and screened for stably transfected cells in MEM with 10% FBS and 400 μg/mL G418. These were called GH cell strains, and they served as a shield control (called “control” in the text and the figures) and basic cell strain for further transfection. A mock group produced by transfecting plasmid pIRES-RFP into GH cell strain served as a mock group. The HCV subgenomic replicon was constructed using two plasmids described in a previous work^17^. p5BR expresses HCV RNA-dependent RNA polymerase NS5B, and prGC3N expresses complementary sequences of HCV *IRES-core* as a HCV RNA subgenomic template. For the HCV subgenomic replicon, GH cells were transfected with plasmid p5BR and identified by detection of HCV NS5B using Western blotting and HCV-*core* levels were assessed using HCV-*core*(+)-sequence-dependent RT-PCR as described in a previous work at a designated time after transfection^17^.

### Cloning and constructs of atg10 and atg10s genes

Autophagy related protein ATG10/ATG10S complete coding sequences were obtained using RT-PCR from a template of HepG2 cDNA1sts with the primer pair (Forward 5′ATGGAAGAAGATGAGTTCATTGG3′ and Reverse 5′TGGCCCTACAATGCTCAGC3′) and inserted into pGEM-T vector. This was followed by sequencing verification. Then eukaryote expression vectors of both *atg10* and *atg10s* were constructed by inserting the two CDS into pIRES2-EGFP for independent protein translation of ATG10/ATG10S and EGFP. These are here designated as pIE-ATG10/ATG10S. Two *rfp*-fused *atg10/atg10s* expressive vectors were produced by inserting *atg10* or *atg10s* CDS into pDsRed-C1 in frame with *dsRed* report gene. These are here designated as pDsRed-ATG10 and pDsRed-atg10s. Transfection and Western blot experiments were used to verify the ATG10/ATG10S overexpression in HepG2 cells. The two fused constructs pDsRed-atg10 and pDsRed-atg10s were used for co-localization experiments.

### Overexpression of ATG10/ATG10S in the HCV subgenomic replicon cells

To assess the action of ATG10 action on HCV subgenomic replicon and autophagy flux, 5′-capped mRNAs of *atg10* and *atg10s* were synthesized *in vitro* using a capped mRNA kit (Ambion, AM1348) with pGEM-T-atg10/atg10s as templates. Each 100 ng 5′-capped mRNA or a mock plasmid was transfected into GH cells for each well (6-well plate) 6 h prior to p5BR. Then the cells were collected after an additional 24–48 h of culture for subsequent experiments.

### Morpholinos and ATG10 downregulation in the HCV subgenomic replicon cells

Morpholino oligomers (sMO and misMO) were obtained from Gene Tools, LLC (Corvalis, OR). The morpholino oligomer sMO targets the *atg10* pre-mRNA intron-3 splicing acceptor site which results in *atg10* downregulation and *atg10S* upregulation. misMO sequence contains five mismatched bases (small letters) as a mock control of sMO. The morpholino oligomer sequences are as below: sMO, 5′-ACAGTCCTGTGTTTCATCACCAAAA-3′ and misMO, 5′-ACAcTCCTcTcTTTgATgACCAAAA-3′. *Atg10* knockdown was carried out by the MOs transfection with concentrations of sMO at 15 pM or 5 pM and of misMO at 15 pM for each well of each 6-well plate in GH cells at 6 h prior to entry of p5BR. Then the cells were cultured for another 24 h and collected for subsequent tests.

### Influence of autophagy inhibitors on ATG10S function in autophagy flux and HCV subreplicon

HepG2 cells was trasfected with Atg10S of 5′ capped RNA; and after 6 hours the cells were again transfected with the plasmid of prGC3N and p5BR. 24 hours later, the cells were exposed to 3-MA (1 mM, Sigma-Aldrich) or CQ (50 μM, Sigma-Aldrich) for 2 days. Then the cells were collected and examined for changes of *HCV-core*, LAMP2, p62 and LC3BII/I using RT-PCR and western blot analysis.

### Constructs and up/down regulation of human IL28A expression

Human *il-28a* cds in pBluescript-II-SK plasmid was synthesized by Sangon Biotech (Shanghai) Co., Ltd. based on the GenBank sequence (No. NM_172138.1). *Egfp*-fused *il28a* expression plasmid was constructed by insertion of *il-28a* into pegfp-C1 vector in frame with egfp open reading frame. The plasmid pIRES2-egfp-il28a was constructed by insertion of *il-28a* into pIRES2-egfp vector. Overexpression of human IL28A was performed by pIRES2-EGFP-IL28A transfection in the GH cells. After 24 h of culture, p5BR was transfected into the cells. The cells were cultivated for another 48 h and collected for subsequent detection. pIRES2-EGFP was used as a mock plasmid in the operation given above. Knockdown of *il28a* was carried out by shRNA (No. 32461-1) transfection and accompanied a mock-shRNA control; the procedure was the same as that used in *il28a* overexpression only with the plasmids replaced by the shRNAs.

### PCR analysis

For semi-quantitative PCR, reverse transcription-PCR was used to assess the ability of the HCV subgenomic replicon and the relative gene expression levels. Total RNA was isolated with Trizol Reagent and divided into two equal parts to reverse transcription with different primers, *Core*-R (5′GCGGAAGCTGGGATGGTCAAAC3′) (for test of the HCV subreplicon) and Oligo dT_18_. The cDNA1st was synthesized from 1 µg of the total RNA using AMV reverse transcriptase (Promega). The target cDNA templates were amplified using PCR with Taq polymerase (TaKaRa, Japan). PCR was performed with primer pairs of related genes, and *β-actin* served as a loading control. The primer sequences are listed in Table 1. Intensity of target bands was quantified by Image Lab software.

**Table 1:** The primers for semi-quantitative PCR assays in the study.

Gene name	Primer sequence
*β-actin*-F	5′AGGGAAATCGTGGGTGACATCAAA3′
*β-actin*-R	5′ACTCATCGTACTCCTGCTTGCTGA3′
*core*-F1	5′TCCTCTTGGCTCTGCTGTC3′
*core*-R2	5′TCACCTTGATGCCGTTCTT 3′
*ifn-a*-F	5′TCGCCCTTTGCTTTACTGA 3′
*ifn-a*-R	5′CCGCATTCATCAGGGGAGT 3′
*ifn-β*-F	5′TGGCAATTGAATGGGAGG 3′
*ifn-β*-R	5′TGGCCTTCAGGTAATGCAG 3′
*il-28*-F	5′ATGAAACTAGACATGACTGGGGACT 3′
*il-28*-R	5′CCTTCGATGTCGACACACAGGTC 3′
*osa1*-F	5′CGGGGAGGGGGTGGAGTTC 3′
*osa1*-R	5′CGCCGGGTCCAGGATCAC 3′
*ddx58*-F	5′CCGGAAGACCCTGGACCCT 3′
*ddx58*-R	5′CCTGCCATCATCCCCTTAG 3′
*irf-3*-F	5′CGGGAGGGATAAGCCAGAC 3′
*irf-3*-R	5′GGCGGCCCCGGTAGA 3′
*irf-7*-F	5′ATGGCCTTGGCTCCTGAG 3′3′
*irf-7*-R	5′CCCGGCTGAGCGCGTACA 3′3′
*tlr-3*-F	5′GCCAGAATTGTGCCAGAAA 3′
*tlr-3*-R	5′AGCCAAGCAAAGGAATCGT 3′
*tlr-7*-F	5′GCCTCCCGCCTAGCTTAC 3′
*tlr-7*-R	5′CGGCGCACAAGGAAATGG 3′
*lamp-1*-F	5′GCCCCTCGCCCTCACC 3′
*lamp-1*-R	5′GCTCCTCCGCGTTGCACTT 3′
*lamp-2*-F	5′TGCCGTTCTCACACTGCTC 3′
*lamp-2*-R	5′CCCAAGGCCACACCCACTG 3′

For quantitative real-time PCR, *HCV-core*, *Il-28*, *tlr7*, *tlr3*. *irf-7*, *irf-3* and *β-actin* were examined by qPCR (Roche 480), the qPCR mix was TransStart Tip Green qPCR SuperMix (Transgen Biotech, China) and parameters were 95 °C 5 min, 95 °C 5 s, 55 °C 15 s, 72 °C 15 s, for 40 cycles. Then the CT values were analyzed and used to prepare graphs according to the instrument manual. The primer sequences for qPCR are listed in Table 2.

**Table 2:** The primers for quantitative Real-time PCR assays in the study.

Gene name	Primer sequence
*tlr-3*-F	5′GCGCTAAAAAGTGAAGAACTGGAT3′
*tlr-3*-R	5′GCTGGACATTGTTCAGAAAGAGG3′
*tlr-7*-F	5′CTTTGGACCTCAGCCACAACCA3′
*tlr-7*-R	5′CGCAACTGGAAGGCATCTTGTAG3′
*irf-3*-F	5′TCTGCCCTCAACCGCAAAGAAG3′
*irf-3*-R	5′TACTGCCTCCACCATTGGTGTC3′
*irf-7*-F	5′CCACGCTATACCATCTACCTGG3′
*irf-7*-R	5′GCTGCTATCCAGGGAAGACACA3′
*Il28*-F	5′TCGCTTCTGCTGAAGGACTGC3′
*Il28*-R	5′CCTCCAGAACCTTCAGCGTCAG3′
*Core*-F	5′CAACCTCGTGGAAGGCGACAAC3′
*Core*-R	5′GGACAGCAGAGCCAAGAGGAAGATAG3′
*β-actin*-F	5′CACCATTGGCAATGAGCGGTTC3′
*β-actin*-R	5′AGGTCTTTGCGGATGTCCACGT3′
*HCV*-F	5′CGGGAGAGCCATAGTGGTCTGCG3′
*HCV-R*	5′CTCGCAAGCACCCTATCAGGCAGTA-3′

### Western blotting and Co-Immunoprecipitation

Briefly, proteins were extracted with RIPA lysis buffer from the treated cells described above and separated in the SDS-PAGE. The protein bands were transferred onto a nitrocellulose membrane by blotting. The membranes were incubated with anti-NS5B, anti-p62, anti-LC3B, anti-LAMP2, anti-TLR3, anti-TLR7, anti-RIG-I, anti-IRF3, anti-IRF7, and anti-β-actin at a dilution range of 1:100 to 1:1000 in TBS containing 1% skim milk. Then the membranes were washed and incubated with HRP-conjugated goat anti-mouse or goat anti-rabbit IgGs (1:1000 dilutions) for 2 h at RT. Proteins were detected using the Supersignal West Pico chemiluminescent substrate (Thermo) with AlphaEase FC Imaging System (Alpha Innotech Corporation). The optical intensities were quantified by Image Lab software.

For immunoprecititation, the procedure followed the Co-Immunoprecipitation Kit Instructions (Thermo SCIENTIFIC). Briefly, cells were harvested and lysed in RIPA lysis buffer. After being pre-binding with protein A/G agarose beads for 1hr at 4 °C, whole-cell lysates were used for immunoprecipitation with the indicated antibodies. Generally, proper amount of designated antibody was added to cell lysates and incubated at 4 °C overnight. Then the mixtures were added protein A/G agarose beads for 1 hr, the immunoprecipitates were extensively washed with PBS and eluted with SDS loading buffer by boiling for 5 min. The co-precipitates were assessed following running SDS-PAGE and West blotting.

### Assessment of co-localization with cellular immunofluorescence

Treated cells were grown on coverslips for 24 h or 48 h and fixed in 4% PBS-buffered paraformaldehyde for 40 min at room temperature. Cells were washed three times with PBS and permeabilized in 3% Triton X-100 for 10 min, then incubated with anti-NS5B, anti-LC3B, anti-p62, or anti-LAMP2 antibodies overnight at 4 °C, and stained with secondary antibodies that matched the corresponding primary antibodies. All secondary antibodies were conjugated with FITC or TRITC (1:100 dilutions). The cells were mounted by ProLong Diamond Antifade Mountant (Invitrogen) containing DAPI dye in mounting medium and observed using Confocal Laser Scanning Microscopy (CLSM, LSM710, Zeiss).

For double co-localization of NS5B-LC3B or NS5B-p62, the HCV subgenomic replicon model cells were fixed and co-stained with two corresponding antibodies, anti-NS5B-FITC-labeled second antibody, and anti-LC3B-/anti-p62-TRITC-labeled second antibody. In the HCV subgenomic replicon and the replicon plus ATG10 or plus ATG10S cells FITC-anti-p62- and TRITC-anti-LC3B antibodies were used for conjunction of p62-cargos with autophagosomes; FITC-anti-LAMP2- and TRITC-anti-p62 antibodies were used for conjunction of p62-cargos with lysosomes; and FITC-anti-NS5B- and TRITC-anti-LAMP2 antibodies were used for indication of HCV subgenomic replicon complex/autophagosomes-lysosomal conjunction. For exploration of IL28A roles in interaction between HCV subgenomic replicon and ATG10S in autophagy flux, GH cells were subjected to triple rounds of transfection. For human IL28A overexpression, pIRES2-egfp-il28a was first transfected into the GH cells and cultured for 18 h. Then *atg10s* mRNA was transfected with 6 h culture, and p5BR was transfected into the cells. The cells grew another 48 h and were fixed for double antibody staining using FITC-anti-LAMP2- and TRITC-anti-p62 antibodies for co-localization of p62-cargos with lysosomes.

Knockdown of *il28a* was carried out by shRNA (No. 32461) transfection with a mock-shRNA control; the procedure was the same as that in *il28a* overexpression above only with the *il28a* shRNA/mock-shRNA replaced pIRES2-egfp-il28*a*. Co-localization of IL28A with ATG10 or with ATG10S was performed with co-transfection of *egfp*-fused *il28a* (pEGFP-C1-*il28a*) and DsRed-fused *atg10/atg10s* (pDsRed-C1-*atg10/atg10s*) plasmids in HepG2 cells and observed at 6, 12, 24, and 48 h post transfection. The immunostains were used with the reporter antibodies, the endogenous IL-28 antibody (ab38570) and ATG10 antibody (sc-70125).

For triple co-localization of lysosomes, IL28A and ATG10S, HepG2 cells were co-transfected two plasmids, pEGFP-C1-il28a and pDsRed-C1-atg10s, and grown for 24 h, followed by lysosomal staining with 2 μM LysoTracker (LysoTracker Yellow HCK-123) at 37 °C for 30 min and replaced with new MEM. The cells were observed and photographed under the CLSM. In order to distinguish lysosomes from the merged yellow GFP and RFP, DAPI staining was omitted and the lysosomal yellow color was adjusted into pseudo blue color late in image processing.

Pearson’s correlation coefficient was applied for identifying co-localization in the confocal images and measured by FIJI software^35^.

### HCV virion infection

Huh7.5 cells were transfected with designed concentrations of ATG10 or ATG10S full-length mRNA using Lipofectamine 2000 (Invitrogen). After 6 hours, the culture supernatants were replaced with fresh complete cultural media, and the transfected cells were then infected with HCV virion (J6/JFH/JC, 45 IU/cell) for 72 hours. Total proteins and RNAs were extracted and detected with WB and qRT-PCR, repectively^36^. For confocal observation of HCV NS5B co-localized with P62, the cells were invaded with the HCV virion (J6/JFH/JC, 45 IU/cell) for 48 hours and then fixed for immunostaining.

### Data and statistical analysis

The values of quantitative data including immunoblot and semi-quantitative PCR are relative magnitude that were normalized with loading control and based on intensity of bands. The means and standard deviations in histograms are derived from three independent experiments. Statistical analyses were performed using one-way ANOVA tests and *P* < 0.05 was considered as significant.

## Electronic supplementary material

Supplementary Information
