# Supplementary material for: Dual Roles of Two Isoforms of Autophagy-related Gene ATG10 in HCV-Subgenomic replicon Mediated Autophagy Flux and Innate Immunity
Source: Sci Rep. 2017 Sep 12;7:11250. doi: 10.1038/s41598-017-11105-3 (PMC5595887; doi:10.1038/s41598-017-11105-3)
Supplement: Supplementary file 1 — Supplementary Information [file 41598_2017_11105_MOESM1_ESM.pdf]

Supplementary Information

Dual Roles of Two Isoforms of Autophagy-related Gene *ATG10* in HCV-Subgenomic replicon Mediated Autophagy Flux and Innate Immunity

Qiong Zhao[1,&], Zhan-Ying Hu[1,&], Jing-Pu Zhang[1,*] Jian-Dong Jiang[2], Yuan-yuan Ma[1], Jian-rui Li[1], Zong-gen Peng[1], Jin-hua Chen[1]

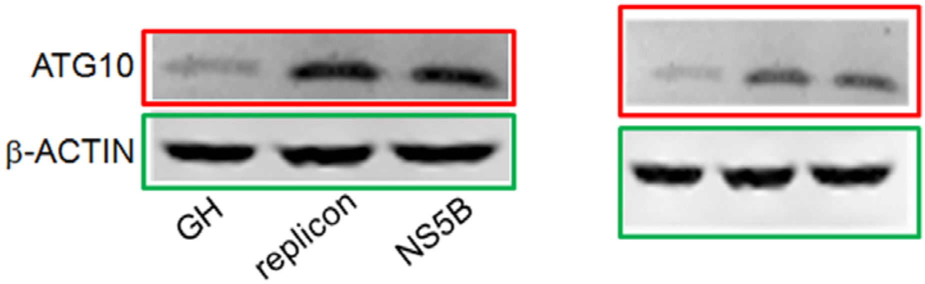

Figure S1. HCV NS5B expression elevated level of ATG10 protein. A NS5B expression plasmid p5BR was transfected into HepG2 cells and ATG10 protein was detected by western blotting, which showed clearly level of ATG10 protein increased by alone NS5B expression as by the replicon compared to GH cell (containing a short HCV subgenomic RNA without NS5B).

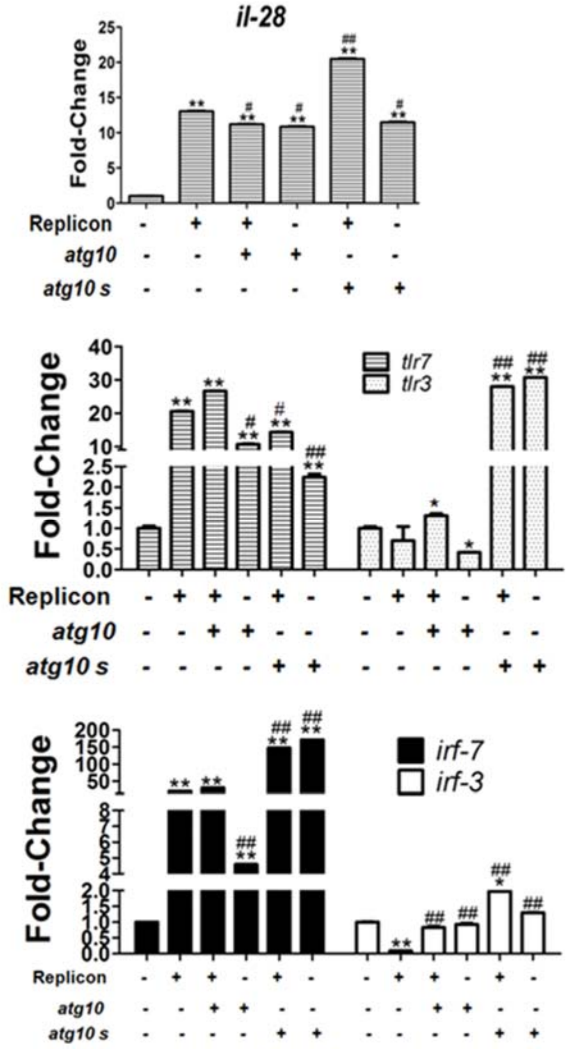

Figure S2. Differential actions of two ATG10 isoforms on the five innate immunologic factors were confirmed by qRT-PCR. The results verify the similar variation of *il28a*, *tlr3*, *tlr7*, *irf-3* and *irf-7* affected by overexpression of atg10 and atg10s to the semi-RT-PCR results (Figure 3) in the text. **P* < 0.05, ***P* < 0.01 *vs* Ctrl; #*P* < 0.05, ##*P* < 0.01 *vs* the replicon.

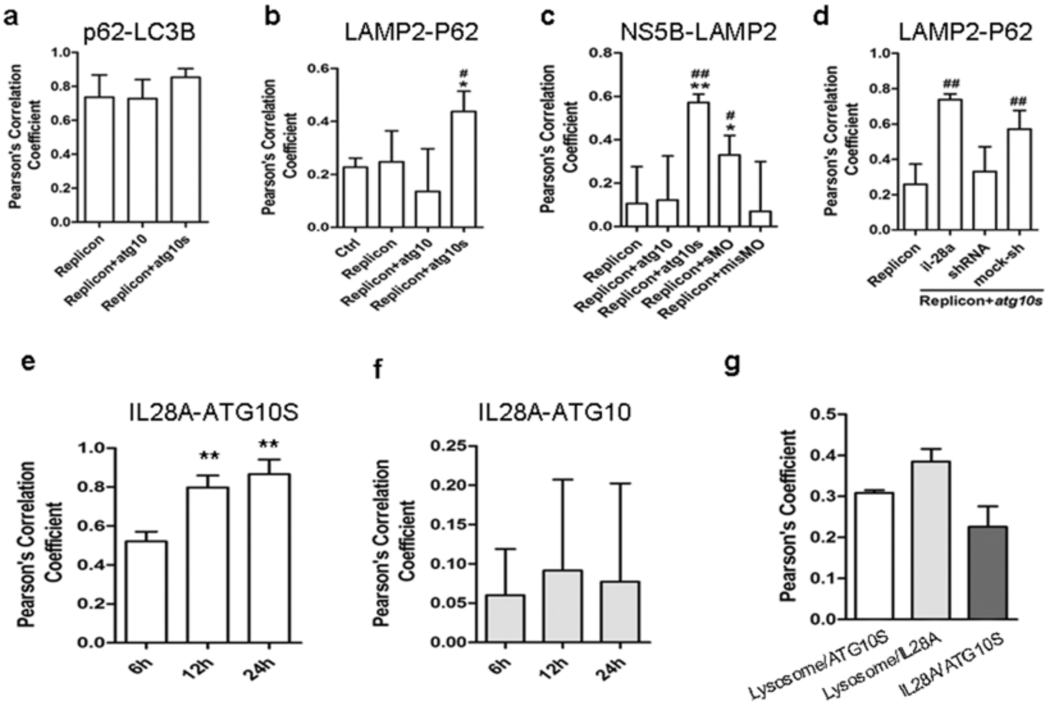

Figure S3. Analysis of Pearson coefficients for colocalization in formation of autophagosomes and autolysosomes regulated by both ATG10s and IL28A.

(a)-(c) The histograms of Pearson coefficients correspond to Figure 4a-4c, respectively. (**a**) The P62-LC3B conjugation shows the similar Pearson coefficients (> 0.7) in the three groups with or without ATG10/ATG10S. (**b**) Pearson coefficient of the LAMP2-P62 conjugation was significantly elevated by ATG10S overexpression but not by ATG10 in the HCV replicon-cells. (**c**) LAMP2-NS5B conjugation was significantly enhanced by ATG10S overexpression or by endogenous ATG10S increase. (**d**) IL28A elevated the Pearson coefficient of the LAMP2-P62 conjugation mediated by ATG10S in the HCV replicon-cells, which corresponds to Figure 5d. (**e**)-(**g**) The histograms of Pearson coefficients correspond to Figure 6a-6c, respectively. (**e**) Pearson coefficient of IL28A-ATG10S combination increased in a time-dependent form in HepG2 cells. (**f**) Pearson coefficient of IL28A-ATG10S combination was not changed by ATG10 in a time-dependent form in HepG2 cells. (**g**) Using lysosome tracker dye, triple colocalization of lysosomes with IL28A and ATG10S was analyzed in HepG2 cells; two-by-two-merged figures show the Pearson coefficients of lysosome-ATG10S, lysosome-IL28A and IL28A-ATG10S in moderate levels in HepG2 cells. * $P < 0.05$, ** $P < 0.01$ *vs* ctrl, replicon+misMO or 6h; # $P < 0.05$, ## $P < 0.01$ *vs* the replicon.

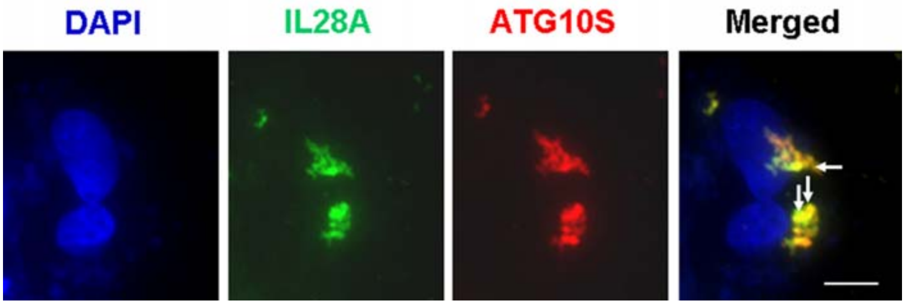

Figure S4. Co-localization of IL28 protein with ATG10S protein was confirmed by the confocal microscopy using their endogenous antibodies. HepG2 cells were co-transfected with pIRES2-EGFP-IL28A and pIRES2-RFP-ATG10S) and cultivated for 24 hr, then were immunostained using IL-28 antibody (ab38570) and ATG10 antibody (sc-70125), which shows the co-localized particle of IL28 with ATG10S (indicated by white arrows).

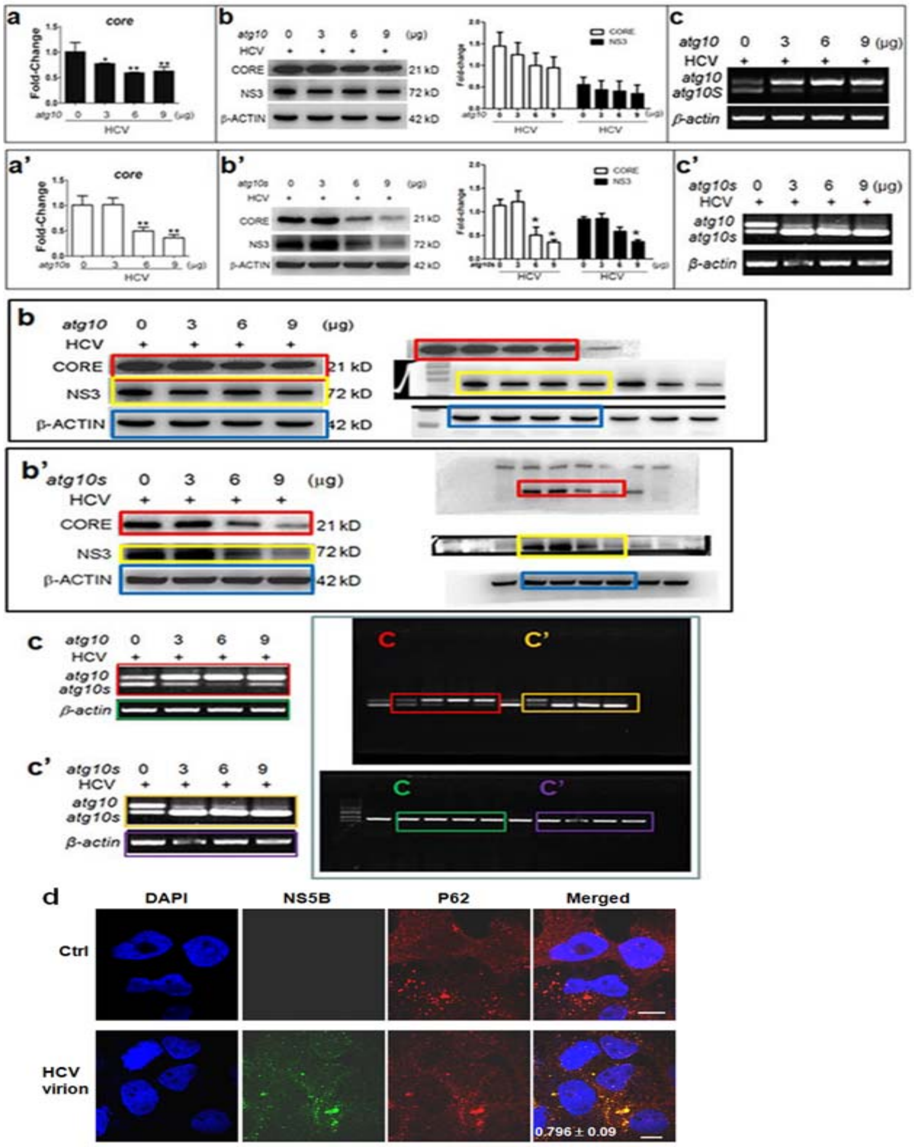

Figure S5. Roles of two isoforms of ATG10 proteins on HCV replication in the HCV-replicating Huh7.5 cell.

(**a, a'**) qRT-PCR results show that HCV core RNA was reduced by overexpression of ATG10 (**a**) and by ATG10S (**a'**), but the ATG10S effect was more powerful than ATG10 and in dose-dependent manner. (**b, b'**) Western blotting results show that levels of HCV CORE and NS3 proteins were decreased slightly by ATG10 (**b**) and significantly by ATG10S in dose-dependent form (**b'**). (**c, c'**) Transcription of ATG10 (**c**) and ATG10S (**c'**) was confirmed using RT-PCR. (**d**) Interaction of NS5B with P62 was examined in Hu 7.5 cells with infective HCV virion by an assessment of co-localization with cellular immunofluorescence, in which co-localized particles of NS5B incorporated with P62 were observed with the value of Pearson correlation coefficient at 0.796 ± 0.09. (Scale bars = 10 μm).

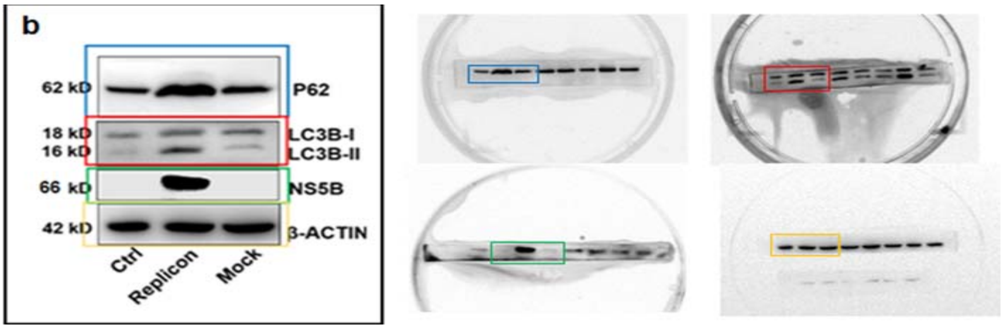

Figure S6b.

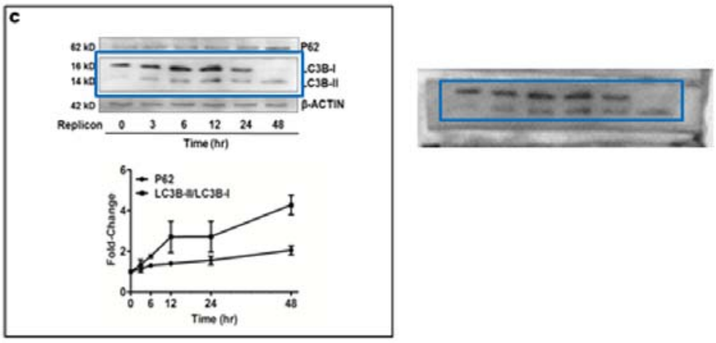

Figure S6c.

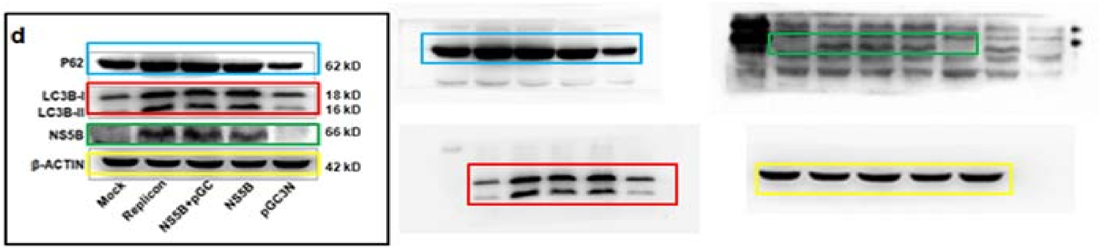

Figure S6d.

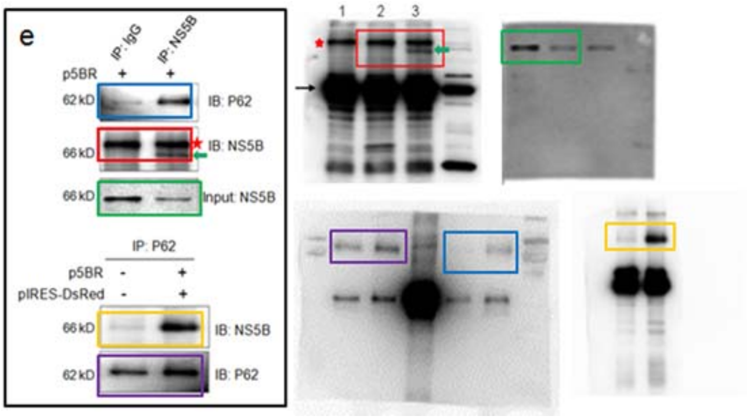

Figure S6e. CO-IP original figures for Figure 1e.

A black arrow shows the positions of IgG heavy chain (about 50 kD), a green arrow indicates NS5B band, and a red star shows non-specific bands. In addition, Lane 1 was loaded protein sample from only pGC3N transfection (no NS5B gene sequence) with IP-antibody of NS5B which did not show NS5B band, Lane 2 was loaded protein sample from only p5BR transfection (an expression construct of HCV-NS5B gene) with IP-antibody of IgG which also did not show NS5B band, Lane 3 was loaded protein sample from pGC3N plus p5BR co-transfection (a minimum HCV subreplicon) with IP-antibody of NS5B which showed the NS5B band (about 66 kD).

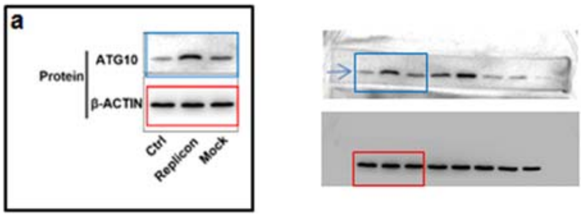

Figure S7a.

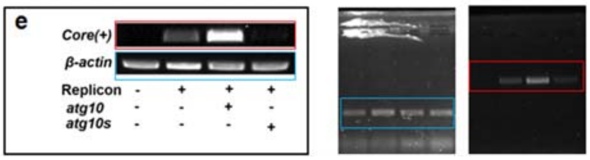

Figure S7e.

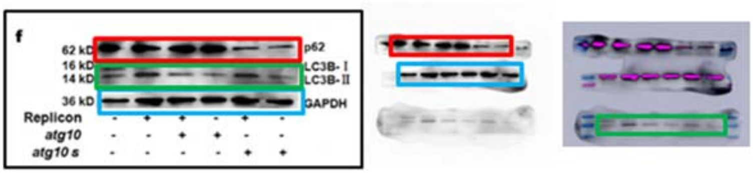

Figure S7f.

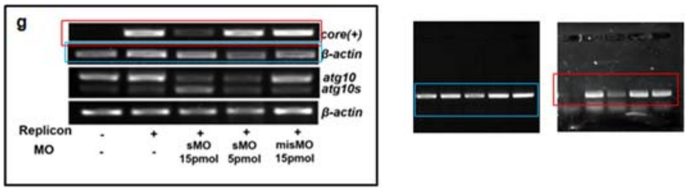

Figure S7g.

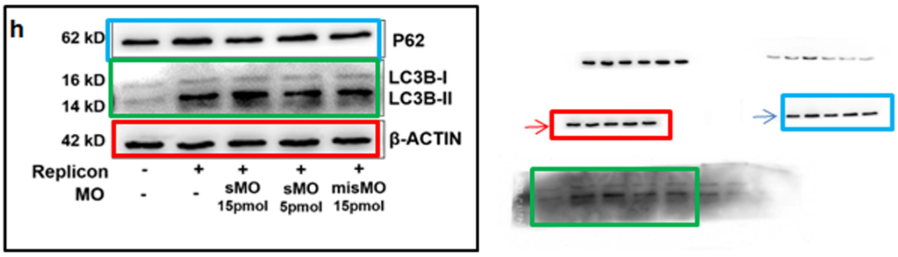

Figure S7h.

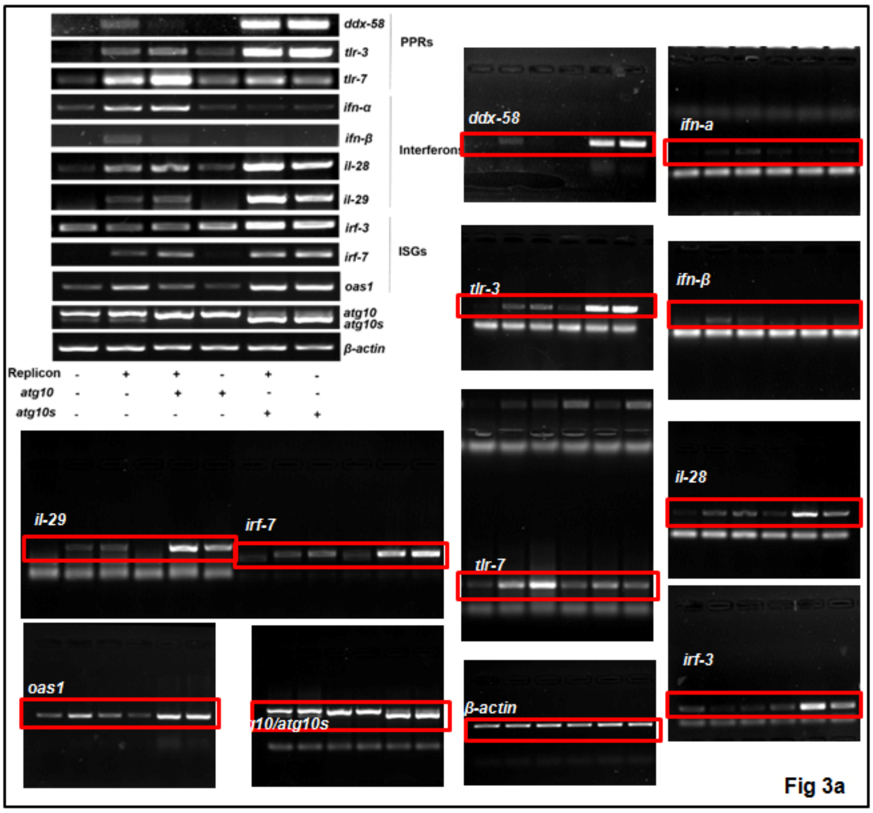

Figure S8a.

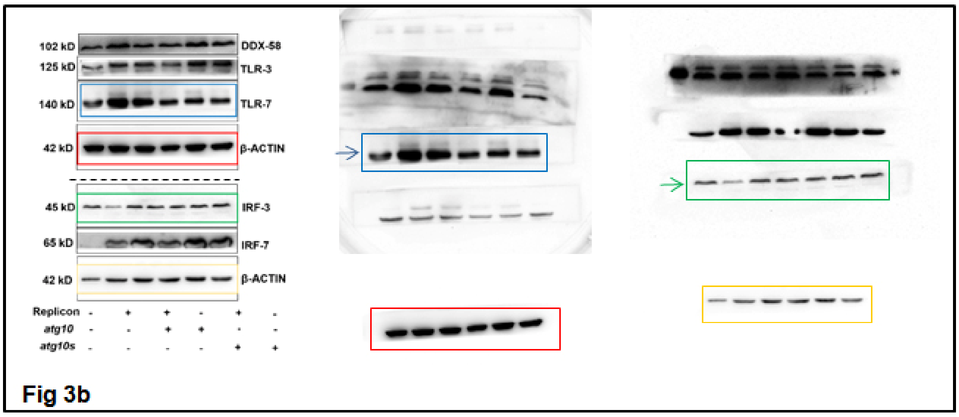

Figure S8b.

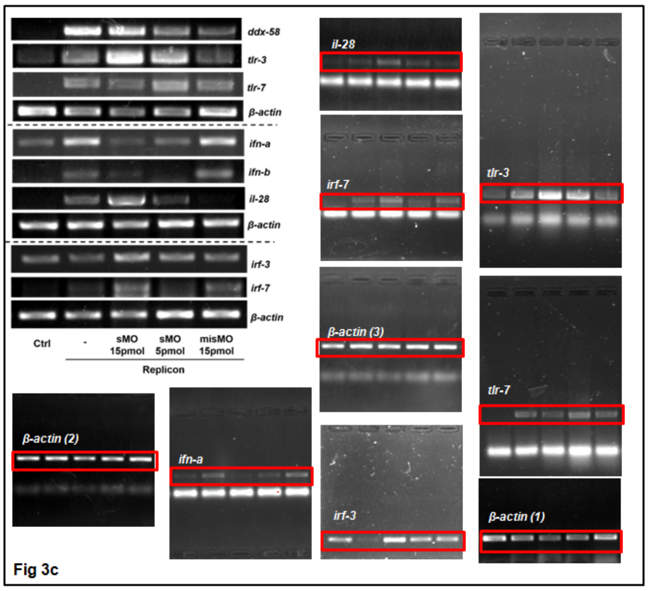

Figure S8c.

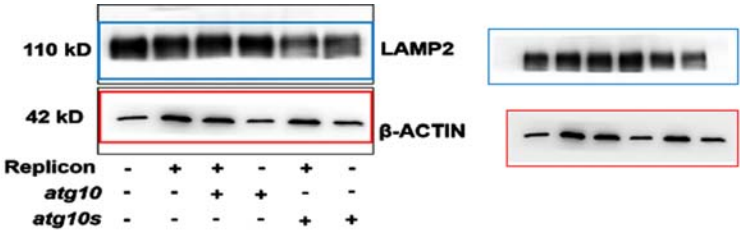

Figure S9d.

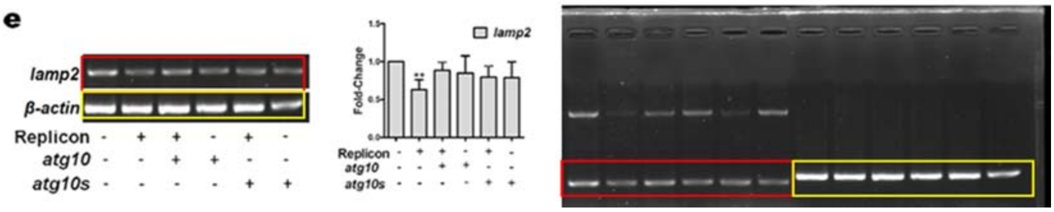

Figure S9e.

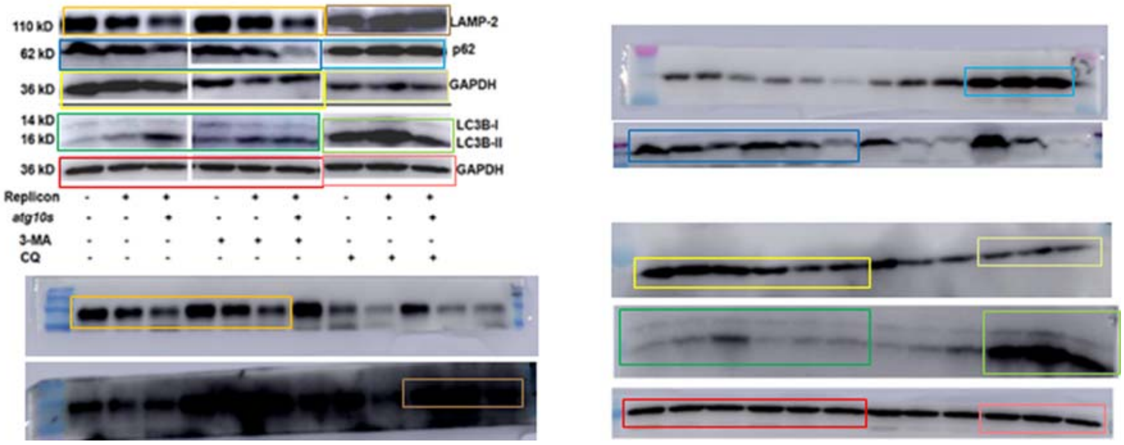

Figure S9f.

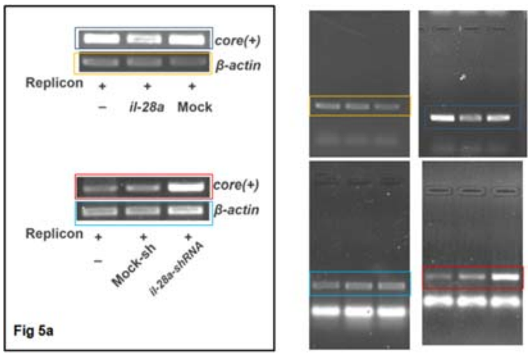

Figure S10a.

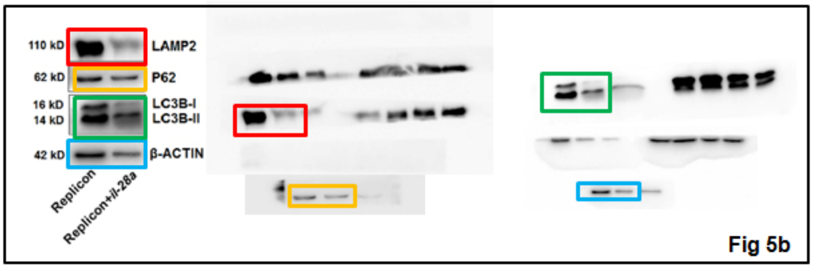

Figure S10b.

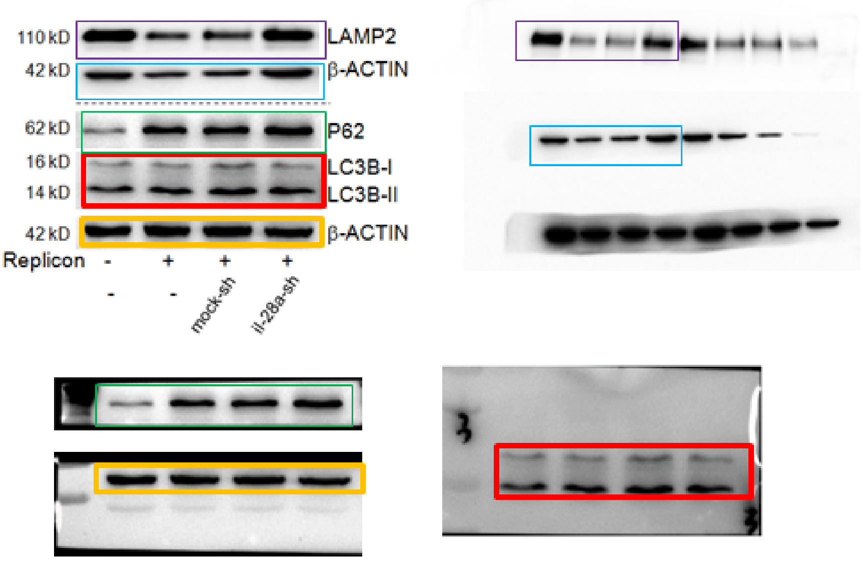

Figure S10c.

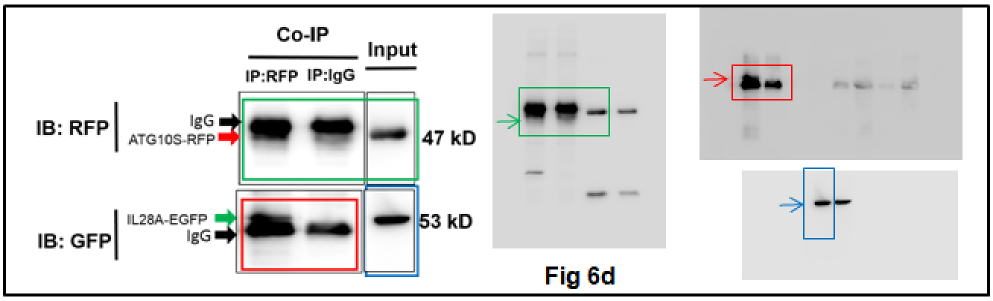

Figure S11d.
